# Supplementary material for: Functional characterization of neuropeptides that act as ligands for both calcitonin-type and pigment-dispersing factor-type receptors in a deuterostome
Source: eLife. 2025 Nov 21;13:RP101799. doi: 10.7554/eLife.101799 (PMC12638048; doi:10.7554/eLife.101799)
Supplement: Figure 1—source data 1. [file elife-101799-fig1-data1.docx]

| Abbreviation | Species Names | Accession Numbers And/or Citations |
| --- | --- | --- |
| *H_sapCTP* | *Homo sapiens* | GI:179819 |
| *H_sapCGRPP* | *Homo sapiens* | NP_001029125.1 |
| *T_rubCTP* | *Takifugu rubripes* | GenBank: CAC81278.1 |
| *T_rubCGRPP* | *Takifugu rubripes* | GenBank: CAC81277.1 |
| *C_intCTP* | *Ciona intestinalis* | GenBank: BAI63095.1 |
| *A_japCTP1* | *Apostichopus japonicus* | GenBank: AWU78769.1 |
| *A_japCTP2* | *Apostichopus japonicus* | GenBank: AWU78770.1 |
| *A_rubCTP* | *Asterias rubens* | GenBank: KT601715.1 |
| *S_purCTP* | *Strongylocentrotus purpuratus* | GI:115767208 |
| *H_scaCTP1* | *Holothuria scabra* | Suwansa-Ard et al. (2018) |
| *H_scaCTP2* | *Holothuria scabra* |  |
| *H_glaCTP1* | *Holothuria glaberrima* |  |
| *H_glaCTP2* | *Holothuria glaberrima* |  |
| *L_gigCTP* | *Lottia gigantea* | GI:163526287 |
| *D_melDH31P* | *Drosophila melanogaster* | NP_523514.1 |
| *L_migDH31P* | *Locusta migratoria* | GenBank: AKN21237.1 |
| *C_telCTP* | *Capitella teleta* | GI: 161220966 |

Accession numbers or citations of calcitonin-type family peptides or precursors in Bilateria

**References:**

Suwansa-Ard S, Chaiyamoon A, Talarovicova A, et al. Transcriptomic discovery and comparative analysis of neuropeptide precursors in sea cucumbers (Holothuroidea). Peptides, 2018, 99: 231-240. doi: 10.1016/j.peptides.2017.10.008.
