# Supplementary material for: Functional characterization of neuropeptides that act as ligands for both calcitonin-type and pigment-dispersing factor-type receptors in a deuterostome
Source: eLife. 2025 Nov 21;13:RP101799. doi: 10.7554/eLife.101799 (PMC12638048; doi:10.7554/eLife.101799)
Supplement: Figure 2—source data 1. [file elife-101799-fig2-data1.docx]

Accession numbers of CT-type, PDF-type and CRH-type receptor families in Bilateria

| Species | Protein ID | Gene code | Species code |
| --- | --- | --- | --- |
| *Amphiura filiformis* | XP_072029112.1 | CRHR | Afil |
| *Amphiura filiformis* | XP_072021544.1 | CTR | Afil |
| *Amphiura filiformis* | XP_072029557.1 | PDFR1 | Afil |
| *Amphiura filiformis* | XP_072028436.1 | PDFR2 | Afil |
| *Anneissia japonica* | XP_033108182.1 | CRHR | Anja |
| *Anneissia japonica* | XP_033104142.1 | CTR | Anja |
| *Anneissia japonica* | XP_033104113.1 | PDFR | Anja |
| *Apis mellifera* | XP_006564466.1 | DH31R | Amel |
| *Apis mellifera* | XP_016768833.2 | PDFR | Amel |
| *Aplysia californica* | XP_012938918.2 | CRHR | Acal |
| *Aplysia californica* | XP_005089839.2 | CTR | Acal |
| *Aplysia californica* | XP_005093780.3 | DH31R | Acal |
| *Apostichopus japonicus* | XP_071811976.1 | CRHR | Ajap |
| *Apostichopus japonicus* | PIK36177.1 | CRHR | Ajap |
| *Apostichopus japonicus* | XP_071811994.1 | CRHR | Ajap |
| *Apostichopus japonicus* | PIK41405.1 | CRHR | Ajap |
| *Apostichopus japonicus* | XP_071812618.1 | CTR | Ajap |
| *Apostichopus japonicus* | XP_071811731.1 | PDFR1 | Ajap |
| *Apostichopus japonicus* | XP_071812243.1 | PDFR2 | Ajap |
| *Asterias rubens* | ENSASRP00000008640.1 | CRHR | Arub |
| *Asterias rubens* | ENSASRP00000013963.1 | CRHR | Arub |
| *Asterias rubens* | ENSASRP00000014061.1 | CRHR | Arub |
| *Asterias rubens* | ENSASRP00000011186.1 | CTR | Arub |
| *Asterias rubens* | ENSASRP00000010309.1 | PDFR1 | Arub |
| *Asterias rubens* | ENSASRP00000010166.1 | PDFR2 | Arub |
| *Bombus impatiens* | ENSBIMP00000009704.1 | CRHR | Bimp |
| *Bombus impatiens* | ENSBIMP00000023653.1 | DH31R | Bimp |
| *Bombus impatiens* | ENSBIMP00000026387.1 | PDFR | Bimp |
| *Branchiostoma floridae* | XP_035687893.1 | CRHR | Bflo |
| *Branchiostoma floridae* | XP_035687894.1 | CRHR | Bflo |
| *Branchiostoma floridae* | XP_035689670.1 | CRHR | Bflo |
| *Branchiostoma floridae* | XP_035689671.1 | CRHR | Bflo |
| *Branchiostoma floridae* | XP_035687895.1 | CTR* | Bflo |
| *Caenorhabditis elegans* | NP_001122749.1 | SEB2* | Cele |
| *Ciona intestinalis* | XP_002122381.1 | CRHR | Cint |
| *Ciona intestinalis* | XP_009858716.1 | CRHR | Cint |
| *Drosophila melanogaster* | NP_725278.1 | DH31R* | Dmel |
| *Drosophila melanogaster* | NP_001285174.1 | HECTOR | Dmel |
| *Drosophila melanogaster* | CAB72288.1 | PDFR | Dmel |
| *Gallus gallus* | ENSGALP00010018855.1 | CLR* | Ggal |
| *Gallus gallus* | ENSGALP00010039362.1 | CRHR | Ggal |
| *Gallus gallus* | ENSGALP00010039367.1 | CRHR | Ggal |
| *Gallus gallus* | ENSGALP00010033414.1 | CRHR | Ggal |
| *Gallus gallus* | ENSGALP00010004560.1 | CTR* | Ggal |
| *Gekko gecko* | ENSEGEP00000030211.1 | CLR | Ggec |
| *Gekko gecko* | ENSEGEP00000005175.1 | CRHR | Ggec |
| *Gekko gecko* | ENSEGEP00000009950.1 | CRHR | Ggec |
| *Gekko gecko* | ENSEGEP00000001898.1 | CTR | Ggec |
| *Homo sapiens* | ENST04850112052.1 | CLR* | Hsap |
| *Homo sapiens* | ENST04850047995.1 | CRHR | Hsap |
| *Homo sapiens* | ENST04850093643.1 | CRHR | Hsap |
| *Homo sapiens* | ENST04850217190.1 | CTR* | Hsap |
| *Hypsibius exemplaris* | OQV19025.1 | CRHR | Hexe |
| *Hypsibius exemplaris* | OQV18662.1 | CRHR | Hexe |
| *Hypsibius exemplaris* | OWA52582.1 | CTR | Hexe |
| *Hypsibius exemplaris* | OWA52579.1 | DH31Ra | Hexe |
| *Hypsibius exemplaris* | OQV24416.1 | DH31Rb | Hexe |
| *Hypsibius exemplaris* | OQV13187.1 | PDFR | Hexe |
| *Lepisosteus oculatus* | XP_015199664.1 | CLRa | Locu |
| *Lepisosteus oculatus* | XP_015214520.1 | CLRb | Locu |
| *Lepisosteus oculatus* | XP_015209811.1 | CRHR | Locu |
| *Lepisosteus oculatus* | XP_015217722.1 | CRHR | Locu |
| *Lepisosteus oculatus* | XP_006636078.2 | CTR | Locu |
| *Limulus polyphemus* | XP_013771881.1 | CRHR | Lpol |
| *Limulus polyphemus* | XP_022240360.1 | CRHR | Lpol |
| *Limulus polyphemus* | XP_013775447.1 | CRHR | Lpol |
| *Limulus polyphemus* | XP_013791145.1 | CRHR | Lpol |
| *Limulus polyphemus* | XP_013791446.1 | CRHR | Lpol |
| *Limulus polyphemus* | XP_022240480.1 | CTRa | Lpol |
| *Limulus polyphemus* | XP_022240470.1 | CTRb | Lpol |
| *Limulus polyphemus* | XP_022254242.1 | CTRc | Lpol |
| *Limulus polyphemus* | XP_022237602.1 | CTRd | Lpol |
| *Limulus polyphemus* | XP_022246293.1 | DH31Ra | Lpol |
| *Limulus polyphemus* | XP_022235154.1 | DH31Rb | Lpol |
| *Limulus polyphemus* | XP_022235431.1 | DH31Rc | Lpol |
| *Limulus polyphemus* | XP_022239928.1 | DH31Rd | Lpol |
| *Limulus polyphemus* | XP_022248891.1 | DH31Re | Lpol |
| *Limulus polyphemus* | XP_022255301.1 | PDFR | Lpol |
| *Lineus longissimus* | ENSLLNP00015009405.1 | CRHR | Llon |
| *Lineus longissimus* | ENSLLNP00015009422.1 | CRHR | Llon |
| *Lineus longissimus* | ENSLLNP00015009434.1 | CRHR | Llon |
| *Lineus longissimus* | ENSLLNP00015009638.1 | CTRa | Llon |
| *Lineus longissimus* | ENSLLNP00015008640.1 | CTRb | Llon |
| *Lineus longissimus* | ENSLLNP00015012550.1 | CTRc | Llon |
| *Lineus longissimus* | ENSLLNP00015013093.1 | DH31R | Llon |
| *Lineus longissimus* | ENSLLNP00015009635.1 | PDFR | Llon |
| *Lumbricus terrestris* | ENSVPPP00000022107.1 | CTRa | Lter |
| *Lumbricus terrestris* | ENSVPPP00000011858.1 | CTRb | Lter |
| *Lumbricus terrestris* | ENSVPPP00000018690.1 | CTRc | Lter |
| *Lumbricus terrestris* | ENSVPPP00000033159.1 | CTRd | Lter |
| *Lumbricus terrestris* | ENSVPPP00000030225.1 | DH31Ra | Lter |
| *Lumbricus terrestris* | ENSVPPP00000022149.1 | DH31Rb | Lter |
| *Lumbricus terrestris* | ENSVPPP00000003533.1 | PDFR | Lter |
| *Magallana gigas* | NP_001292279.1 | CTR1* | Mgig |
| *Magallana gigas* | XP_034310675.2 | CTR2* | Mgig |
| *Magallana gigas* | XP_011442634.2 | CTR3 | Mgig |
| *Magallana gigas* | XP_011442636.3 | CTR4 | Mgig |
| *Magallana gigas* | XP_065929766.1 | CTR5 | Mgig |
| *Magallana gigas* | XP_065923133.1 | CTR6 | Mgig |
| *Magallana gigas* | XP_052684037.1 | CTR7 | Mgig |
| *Magallana gigas* | XP_034317823.1 | PDFR | Mgig |
| *Magallana gigas* | XP_034310400.1 |  | Mgig |
| *Owenia fusiformis* | CAC9666549.1 | CRHR | Ofus |
| *Owenia fusiformis* | CAC9666335.1 | CTRa | Ofus |
| *Owenia fusiformis* | CAC9673671.1 | CTRb | Ofus |
| *Owenia fusiformis* | CAC9666125.1 | DH31R | Ofus |
| *Owenia fusiformis* | CAC9578850.1 | PDFR | Ofus |
| *Paramacrobiotus metropolita* | XP_055352663.1 | CRHR | Pmet |
| *Paramacrobiotus metropolita* | XP_055352664.1 | CRHR | Pmet |
| *Paramacrobiotus metropolita* | XP_055329231.1 | CTR | Pmet |
| *Paramacrobiotus metropolita* | XP_055329233.1 | DH31R | Pmet |
| *Paramacrobiotus metropolita* | XP_055331530.1 | PDFR | Pmet |
| *Pecten maximus* | XP_033762144.1 | CRHR | Pmax |
| *Pecten maximus* | XP_033762006.1 | CTRa | Pmax |
| *Pecten maximus* | XP_033762916.1 | CTRb | Pmax |
| *Pecten maximus* | XP_033762874.1 | CTRc | Pmax |
| *Pecten maximus* | XP_033762263.1 | CTRd | Pmax |
| *Pecten maximus* | XP_033762155.1 | DH31Ra | Pmax |
| *Pecten maximus* | XP_033762946.1 | DH31Rb | Pmax |
| *Pecten maximus* | XP_033729115.1 | DH31Rc | Pmax |
| *Pecten maximus* | XP_033762294.1 | PDFR | Pmax |
| *Platynereis dumerilii* | AKQ63006.1 | DH31R-1* | Pdum |
| *Platynereis dumerilii* | WOE99345.1 | PDFR | Pdum |
| *Priapulus caudatus* | XP_014664111.1 | DH31Ra | Pcau |
| *Priapulus caudatus* | XP_014661438.1 | DH31Rb | Pcau |
| *Rotaria socialis* | CAF3356201.1 | PDFRa | Rsoc |
| *Rotaria socialis* | CAF3546535.1 | PDFRb | Rsoc |
| *Saccoglossus kowalevskii* | NP_001161520.1 | CRHR | Skow |
| *Saccoglossus kowalevskii* | XP_006818813.1 | CTR | Skow |
| *Saccoglossus kowalevskii* | XP_006825104.1 | PDFRa | Skow |
| *Saccoglossus kowalevskii* | XP_006825103.1 | PDFRb | Skow |
| *Schmidtea mediterranea* | SMEST051050001 | CRHR | Smed |
| *Schmidtea mediterranea* | SMEST064388001 | CRHR | Smed |
| *Schmidtea mediterranea* | SMEST049048003 | CRHR | Smed |
| *Schmidtea mediterranea* | SMEST080399003 | CTRa | Smed |
| *Schmidtea mediterranea* | SMEST042095002 | CTRb | Smed |
| *Schmidtea mediterranea* | SMEST006966001 | DH31Ra | Smed |
| *Schmidtea mediterranea* | SMEST027375002 | DH31Rb | Smed |
| *Schmidtea mediterranea* | SMEST023013001 | DH31Rc | Smed |
| *Schmidtea mediterranea* | SMEST035956002 | PDFR | Smed |
| *Sepioteuthis lessoniana* | ENSUKOP00000023564.1 | CRHR | Sles |
| *Sepioteuthis lessoniana* | ENSUKOP00000009609.1 | CTRa | Sles |
| *Sepioteuthis lessoniana* | ENSUKOP00000023956.1 | CTRc | Sles |
| *Sepioteuthis lessoniana* | ENSUKOP00000023961.1 | CTRd | Sles |
| *Sepioteuthis lessoniana* | ENSUKOP00000005365.1 | DH31Ra | Sles |
| *Sepioteuthis lessoniana* | ENSUKOP00000007960.1 | DH31Rb | Sles |
| *Sepioteuthis lessoniana* | ENSUKOP00000030254.1 | PDFR | Sles |
| *Strongylocentrotus purpuratus* | XP_003725174.2 | CRHR | Spur |
| *Strongylocentrotus purpuratus* | XP_030845739.1 | CRHR | Spur |
| *Strongylocentrotus purpuratus* | XP_030846612.1 | CRHR | Spur |
| *Strongylocentrotus purpuratus* | XP_030846621.1 | CTR | Spur |
| *Strongylocentrotus purpuratus* | XP_030845952.1 | PDFR1 | Spur |
| *Strongylocentrotus purpuratus* | XP_030845953.1 | PDFR2 | Spur |
| *Styela clava* | XP_039255657.1 | CRHR | Scla |
| *Tribolium castaneum* | XP_008198347.1 | CRHR | Tcas |
| *Tribolium castaneum* | XP_008192711.1 | CRHR | Tcas |
| *Tribolium castaneum* | XP_064212169.1 | CTR | Tcas |
| *Tribolium castaneum* | XP_008193920.1 | DH31Ra | Tcas |
| *Tribolium castaneum* | XP_064210831.1 | DH31Rb | Tcas |
| *Tribolium castaneum* | XP_064212793.1 | PDFR | Tcas |
| *Xenopus tropicalis* | ENSXETP00000114756.1 | CLR | Xtro |
| *Xenopus tropicalis* | ENSXETP00000109962.1 | CRHR | Xtro |
| *Xenopus tropicalis* | ENSXETP00000101014.1 | CRHR | Xtro |
| *Xenopus tropicalis* | ENSXETP00000060302.3 | CTR | Xtro |
