# Supplementary figures and images for: Functional characterization of neuropeptides that act as ligands for both calcitonin-type and pigment-dispersing factor-type receptors in a deuterostome
Source: eLife. 2025 Nov 21;13:RP101799. doi: 10.7554/eLife.101799 (PMC12638048; doi:10.7554/eLife.101799)

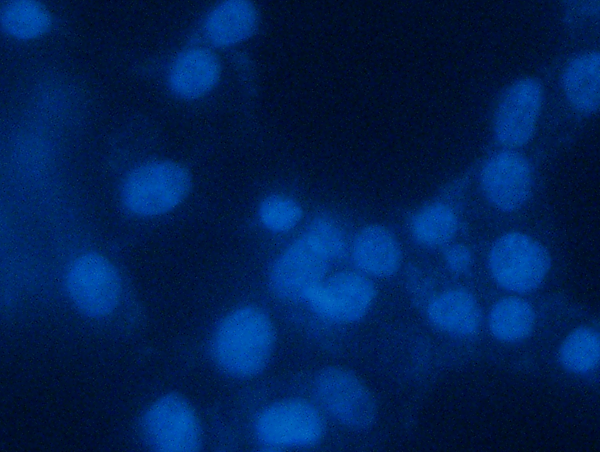

Supplement: Figure 4—source data 1. [file elife-101799-fig4-data1.zip › Figure 4–Source data/Figure 4–Source data 2/AjCTR-AjCT1-DAPI.tif]

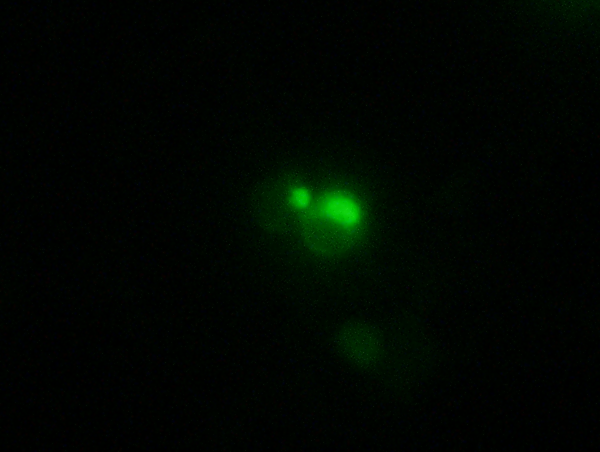

Supplement: Figure 4—source data 1. [file elife-101799-fig4-data1.zip › Figure 4–Source data/Figure 4–Source data 2/AjCTR-AjCT1.tif]

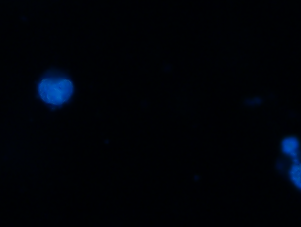

Supplement: Figure 4—source data 1. [file elife-101799-fig4-data1.zip › Figure 4–Source data/Figure 4–Source data 2/AjCTR-AjCT2-DAPI.tif]

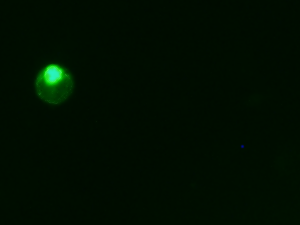

Supplement: Figure 4—source data 1. [file elife-101799-fig4-data1.zip › Figure 4–Source data/Figure 4–Source data 2/AjCTR-AjCT2.tif]

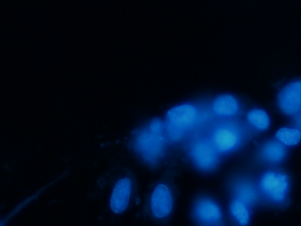

Supplement: Figure 4—source data 1. [file elife-101799-fig4-data1.zip › Figure 4–Source data/Figure 4–Source data 2/AjPDFR1-AjCT1-DAPI.tif]

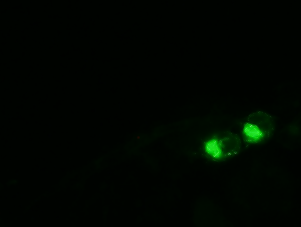

Supplement: Figure 4—source data 1. [file elife-101799-fig4-data1.zip › Figure 4–Source data/Figure 4–Source data 2/AjPDFR1-AjCT1.tif]

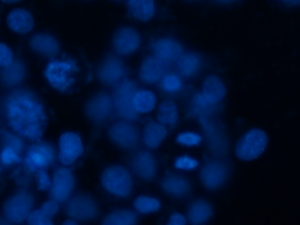

Supplement: Figure 4—source data 1. [file elife-101799-fig4-data1.zip › Figure 4–Source data/Figure 4–Source data 2/AjPDFR1-AjCT2-DAPI.tif]

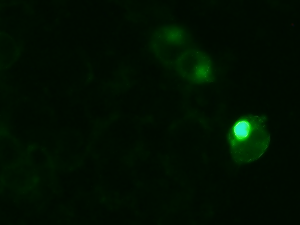

Supplement: Figure 4—source data 1. [file elife-101799-fig4-data1.zip › Figure 4–Source data/Figure 4–Source data 2/AjPDFR1-AjCT2.tif]

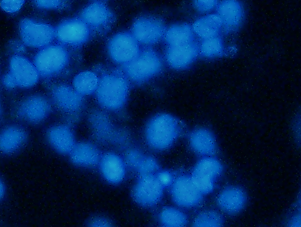

Supplement: Figure 4—source data 1. [file elife-101799-fig4-data1.zip › Figure 4–Source data/Figure 4–Source data 2/AjPDFR2-AjCT1-DAPI.tif]

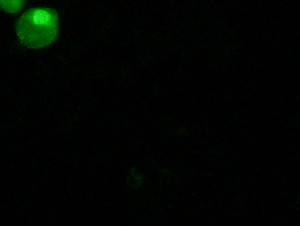

Supplement: Figure 4—source data 1. [file elife-101799-fig4-data1.zip › Figure 4–Source data/Figure 4–Source data 2/AjPDFR2-AjCT1.tif]

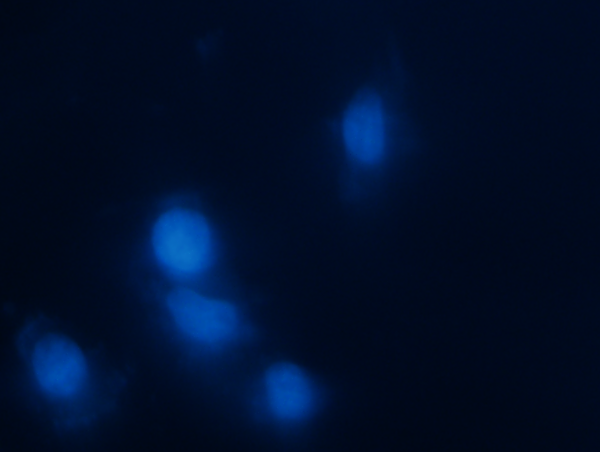

Supplement: Figure 4—source data 1. [file elife-101799-fig4-data1.zip › Figure 4–Source data/Figure 4–Source data 2/AjPDFR2-AjCT2-DAPI.tif]

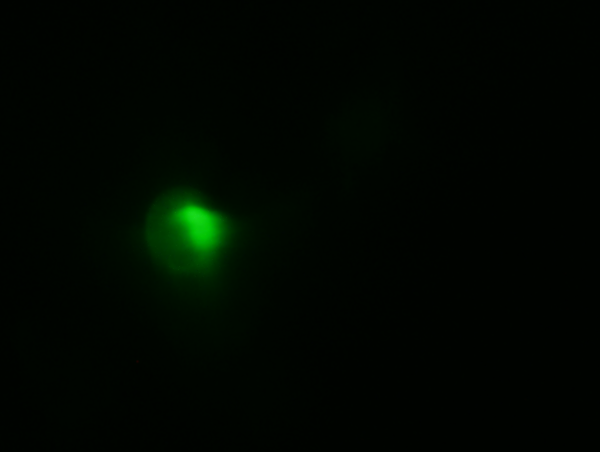

Supplement: Figure 4—source data 1. [file elife-101799-fig4-data1.zip › Figure 4–Source data/Figure 4–Source data 2/AjPDFR2-AjCT2.tif]

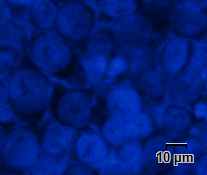

Supplement: Figure 4—source data 2. [file elife-101799-fig4-data2.zip › Figure 4-figure supplement 1-Source data1/AjCTR-serum-free DAPI.tif]

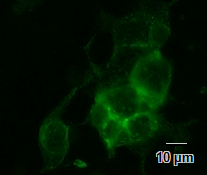

Supplement: Figure 4—source data 2. [file elife-101799-fig4-data2.zip › Figure 4-figure supplement 1-Source data1/AjCTR-serum-free DMEM.tif]

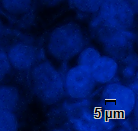

Supplement: Figure 4—source data 2. [file elife-101799-fig4-data2.zip › Figure 4-figure supplement 1-Source data1/AjPDFR1-serum-free DAPI.tif]

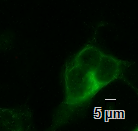

Supplement: Figure 4—source data 2. [file elife-101799-fig4-data2.zip › Figure 4-figure supplement 1-Source data1/AjPDFR1-serum-free DMEM.tif]

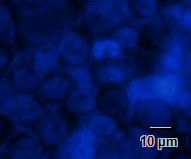

Supplement: Figure 4—source data 2. [file elife-101799-fig4-data2.zip › Figure 4-figure supplement 1-Source data1/AjPDFR2-serum-free DAPI.tif]

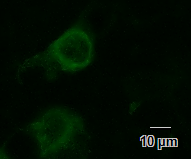

Supplement: Figure 4—source data 2. [file elife-101799-fig4-data2.zip › Figure 4-figure supplement 1-Source data1/AjPDFR2-serum-free DMEM.tif]

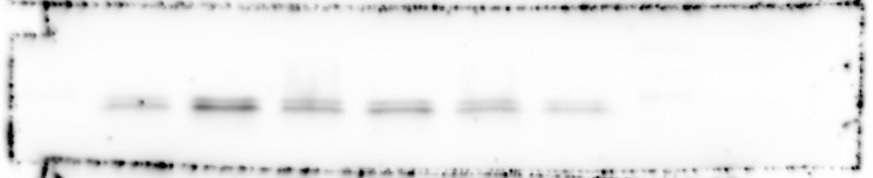

Supplement: Figure 5—source data 3. [file elife-101799-fig5-data3.zip › Figure 5-Source data3/AjCTR-AjCT1-pERK.tif]

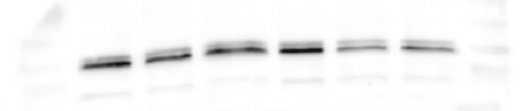

Supplement: Figure 5—source data 3. [file elife-101799-fig5-data3.zip › Figure 5-Source data3/AjCTR-AjCT1-tERK.tif]

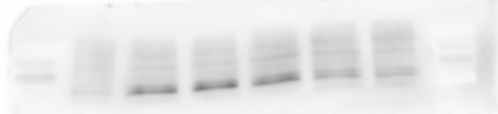

Supplement: Figure 5—source data 3. [file elife-101799-fig5-data3.zip › Figure 5-Source data3/AjCTR-AjCT2-pERK.tif]

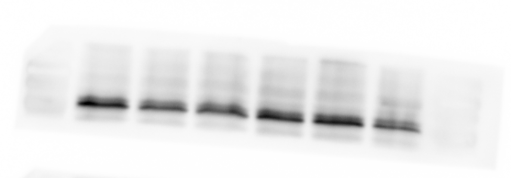

Supplement: Figure 5—source data 3. [file elife-101799-fig5-data3.zip › Figure 5-Source data3/AjCTR-AjCT2-tERK.tif]

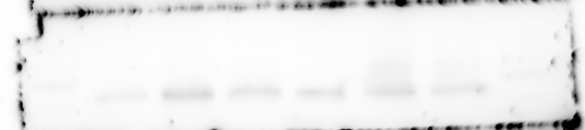

Supplement: Figure 5—source data 3. [file elife-101799-fig5-data3.zip › Figure 5-Source data3/AjPDFR1-AjCT1-pERK.tif]

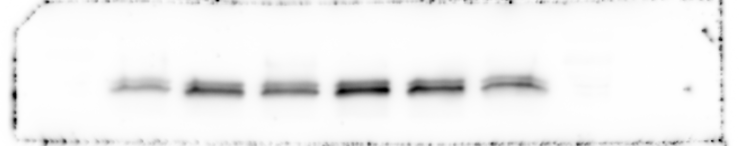

Supplement: Figure 5—source data 3. [file elife-101799-fig5-data3.zip › Figure 5-Source data3/AjPDFR1-AjCT1-tERK.tif]

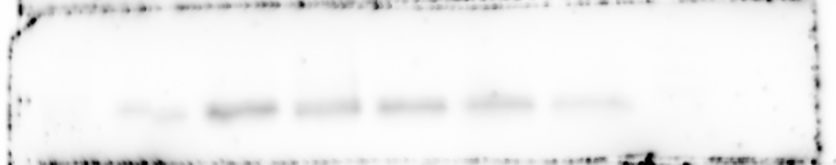

Supplement: Figure 5—source data 3. [file elife-101799-fig5-data3.zip › Figure 5-Source data3/AjPDFR1-AjCT2-pERK.tif]

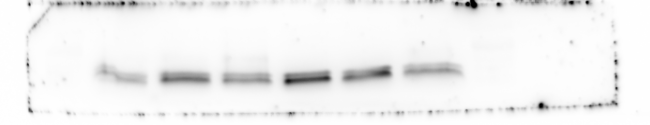

Supplement: Figure 5—source data 3. [file elife-101799-fig5-data3.zip › Figure 5-Source data3/AjPDFR1-AjCT2-tERK.tif]

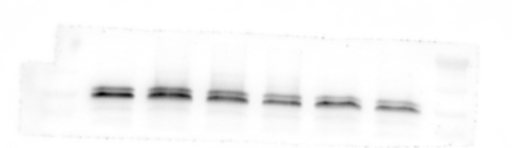

Supplement: Figure 5—source data 3. [file elife-101799-fig5-data3.zip › Figure 5-Source data3/AjPDFR2-AjCT1-pERK.tif]

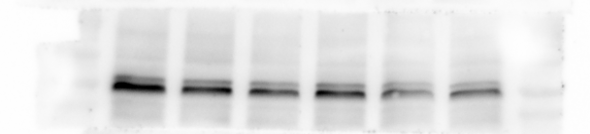

Supplement: Figure 5—source data 3. [file elife-101799-fig5-data3.zip › Figure 5-Source data3/AjPDFR2-AjCT1-tERK.tif]

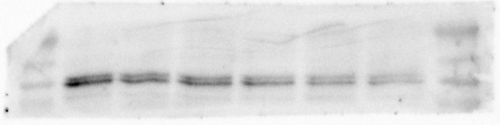

Supplement: Figure 5—source data 3. [file elife-101799-fig5-data3.zip › Figure 5-Source data3/AjPDFR2-AjCT2-pERK.tif]

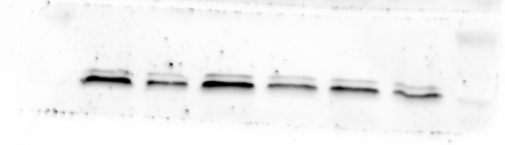

Supplement: Figure 5—source data 3. [file elife-101799-fig5-data3.zip › Figure 5-Source data3/AjPDFR2-AjCT2-tERK.tif]

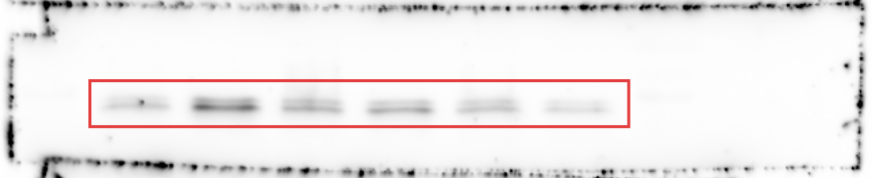

Supplement: Figure 5—source data 4. [file elife-101799-fig5-data4.zip › Figure 5-Source data3/AjCTR-AjCT1-pERK.tif]

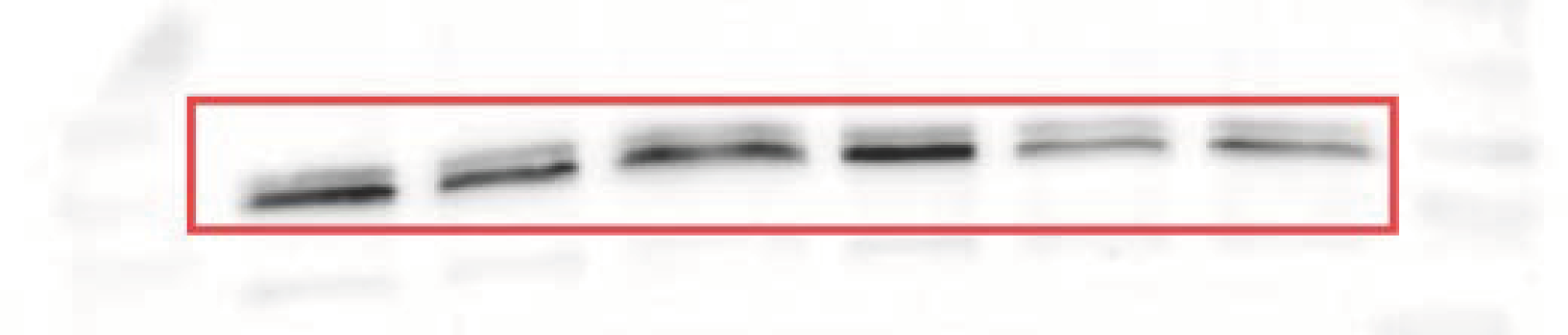

Supplement: Figure 5—source data 4. [file elife-101799-fig5-data4.zip › Figure 5-Source data3/AjCTR-AjCT1-tERK.tif]

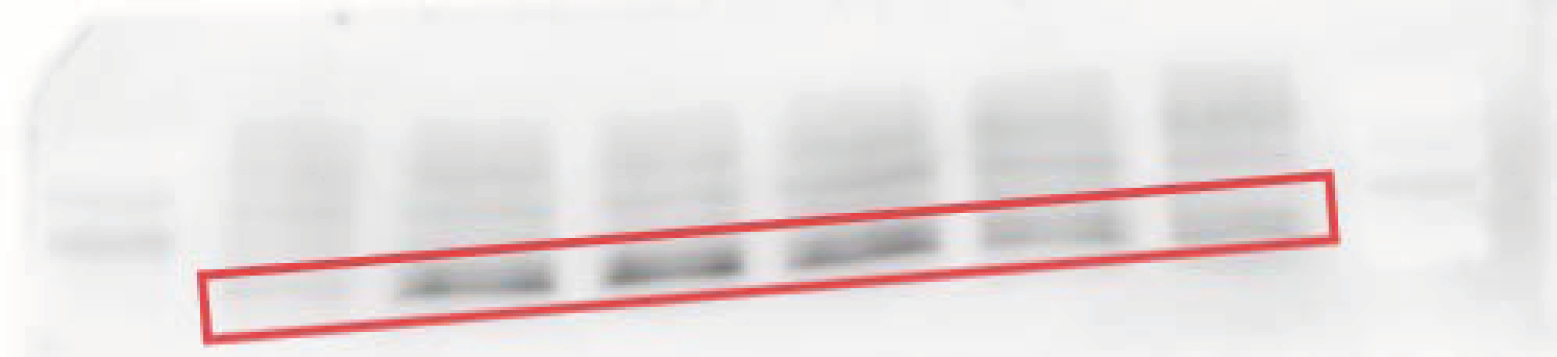

Supplement: Figure 5—source data 4. [file elife-101799-fig5-data4.zip › Figure 5-Source data3/AjCTR-AjCT2-pERK.tif]

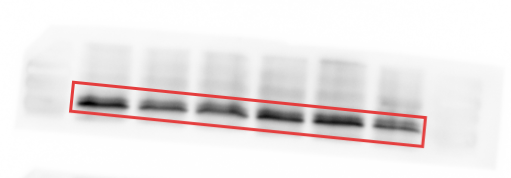

Supplement: Figure 5—source data 4. [file elife-101799-fig5-data4.zip › Figure 5-Source data3/AjCTR-AjCT2-tERK.tif]

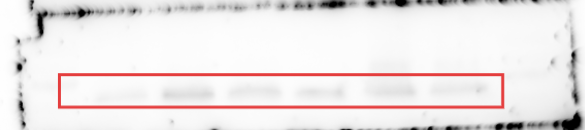

Supplement: Figure 5—source data 4. [file elife-101799-fig5-data4.zip › Figure 5-Source data3/AjPDFR1-AjCT1-pERK.tif]

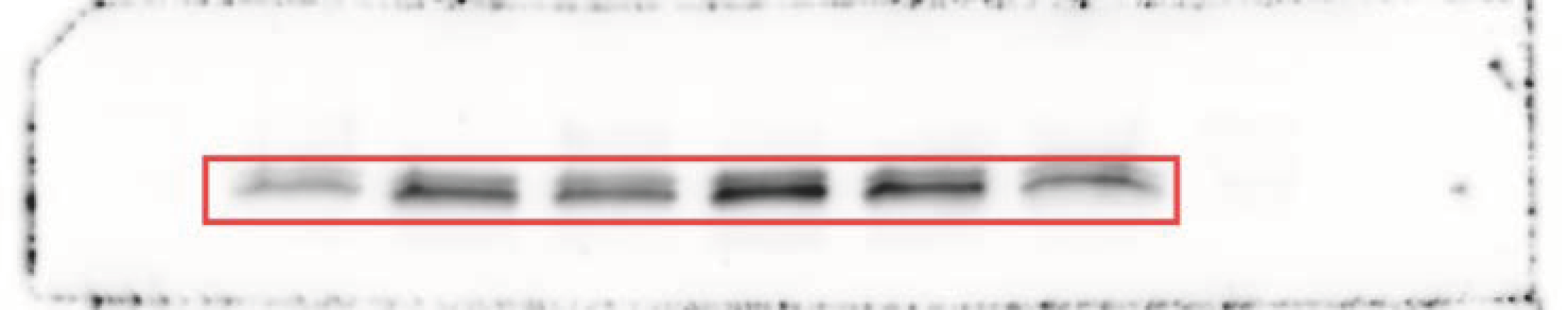

Supplement: Figure 5—source data 4. [file elife-101799-fig5-data4.zip › Figure 5-Source data3/AjPDFR1-AjCT1-tERK.tif]

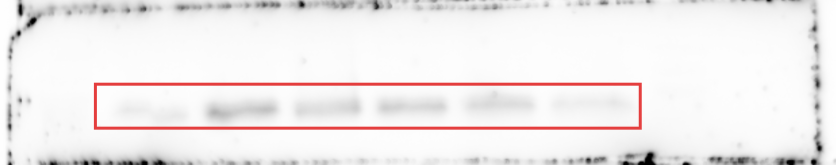

Supplement: Figure 5—source data 4. [file elife-101799-fig5-data4.zip › Figure 5-Source data3/AjPDFR1-AjCT2-pERK.tif]

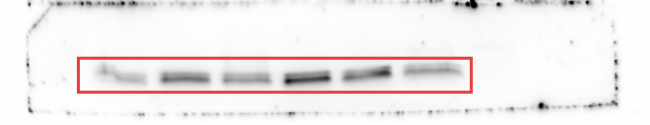

Supplement: Figure 5—source data 4. [file elife-101799-fig5-data4.zip › Figure 5-Source data3/AjPDFR1-AjCT2-tERK.tif]

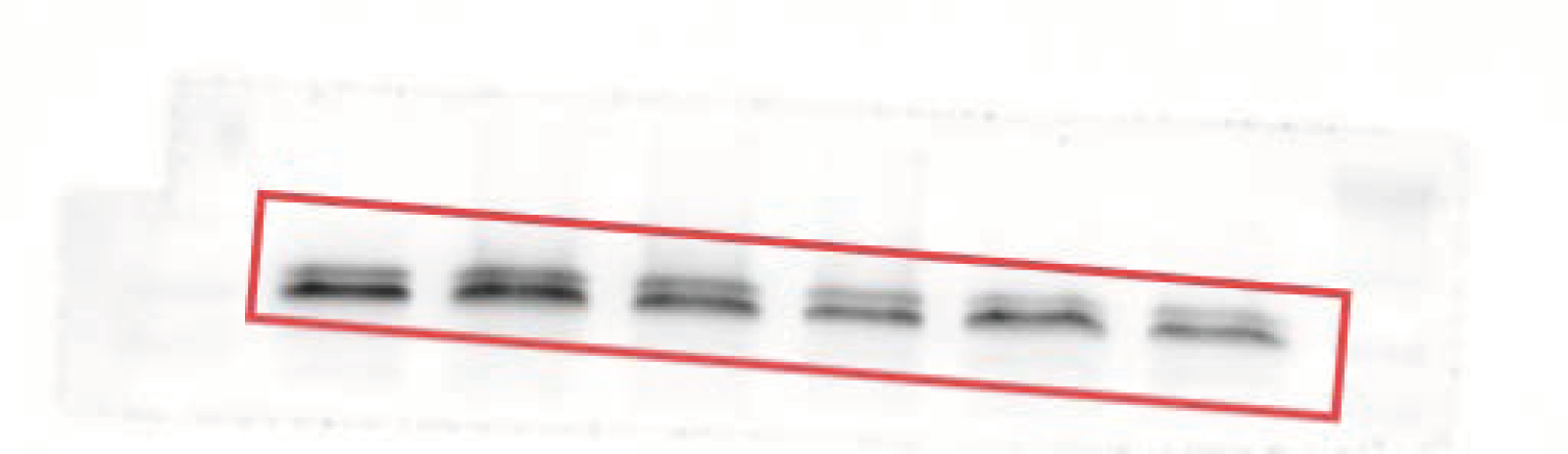

Supplement: Figure 5—source data 4. [file elife-101799-fig5-data4.zip › Figure 5-Source data3/AjPDFR2-AjCT1-pERK.tif]

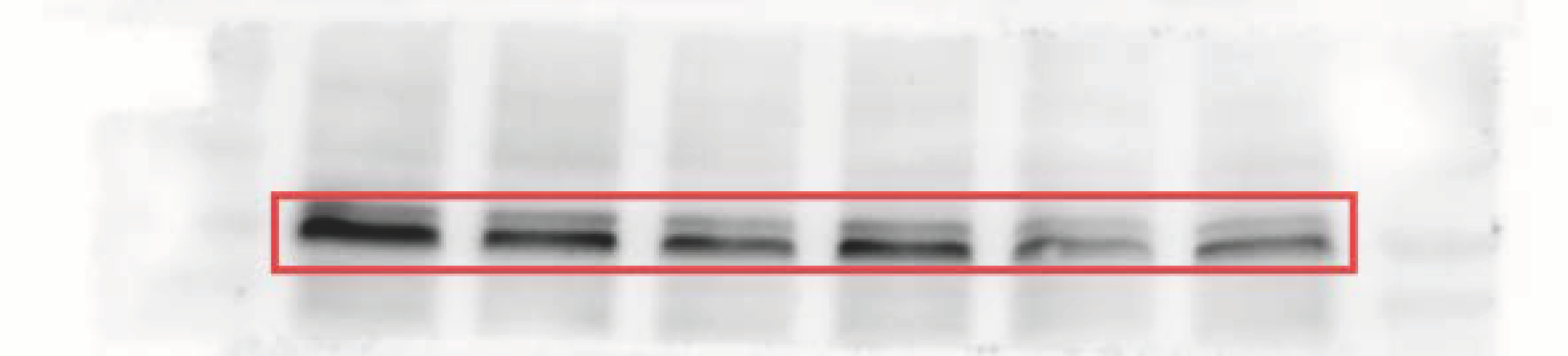

Supplement: Figure 5—source data 4. [file elife-101799-fig5-data4.zip › Figure 5-Source data3/AjPDFR2-AjCT1-tERK.tif]

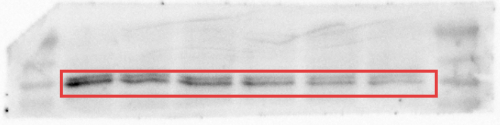

Supplement: Figure 5—source data 4. [file elife-101799-fig5-data4.zip › Figure 5-Source data3/AjPDFR2-AjCT2-pERK.tif]

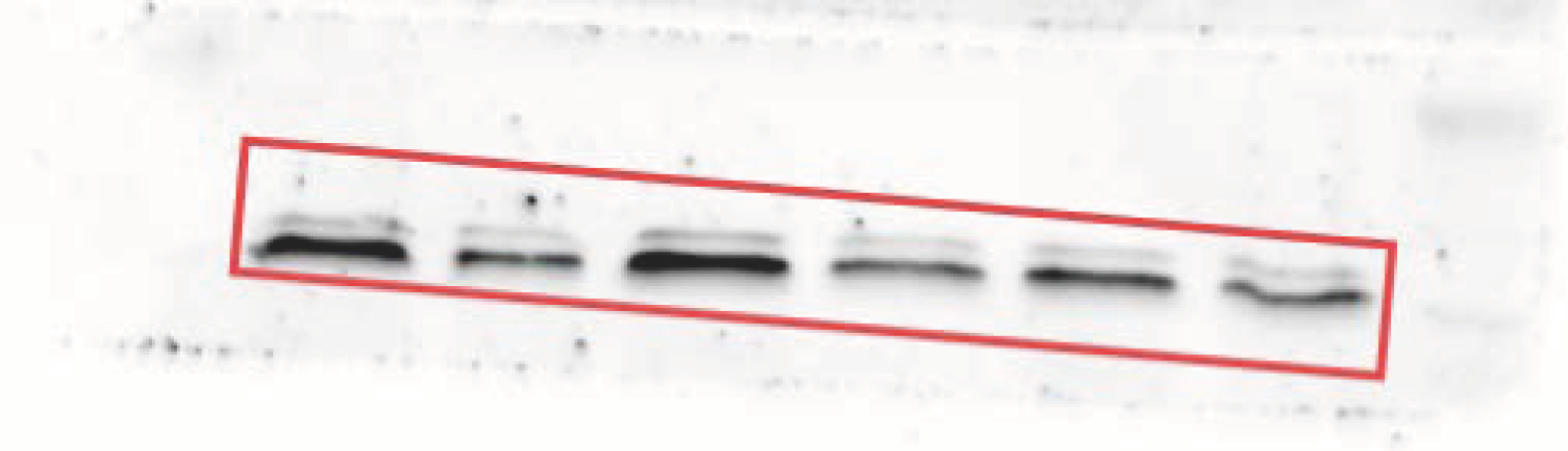

Supplement: Figure 5—source data 4. [file elife-101799-fig5-data4.zip › Figure 5-Source data3/AjPDFR2-AjCT2-tERK.tif]

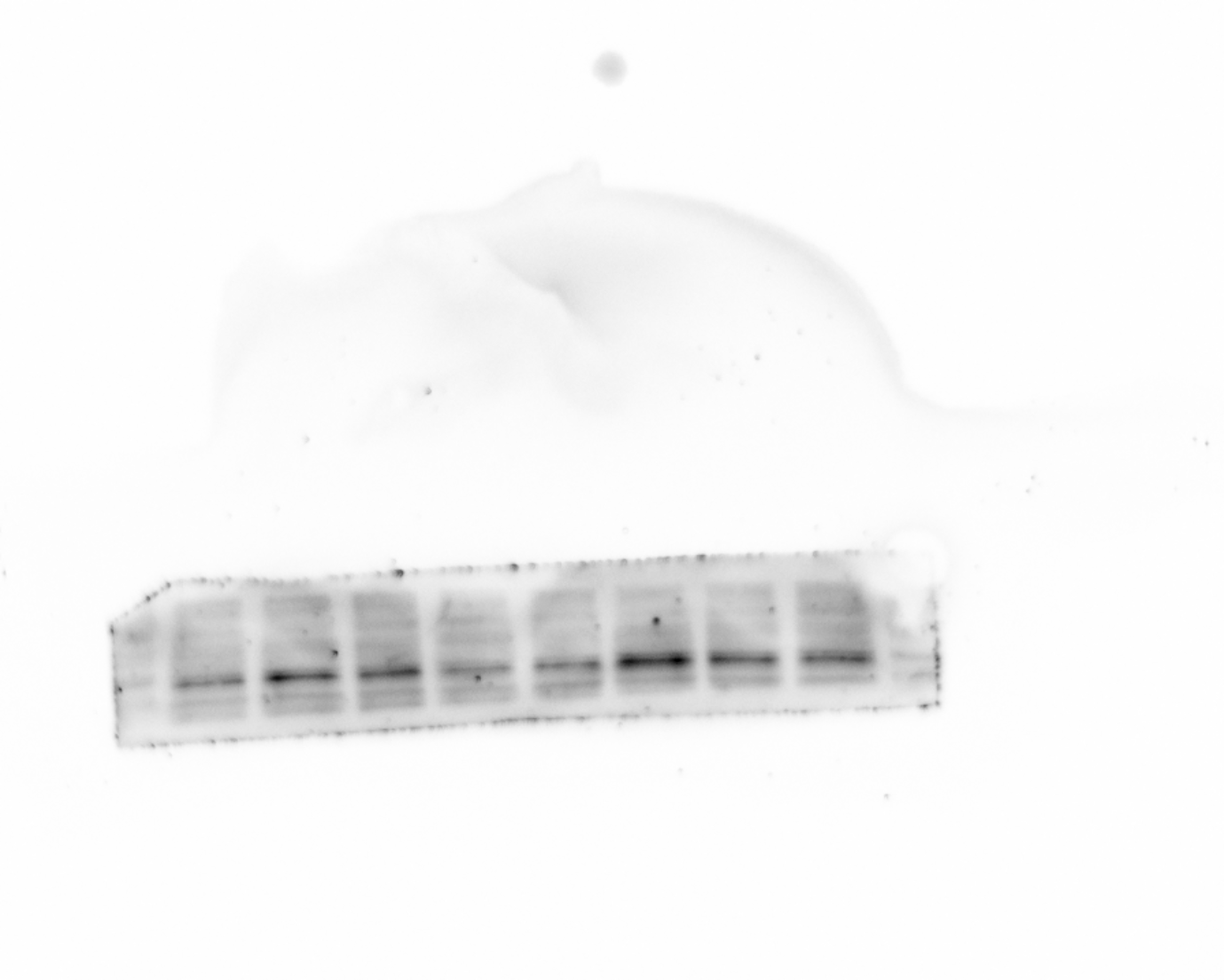

Supplement: Figure 5—figure supplement 1—source data 3. [file elife-101799-fig5-figsupp1-data3.zip › Figure 5-figure supplement 1-1/AjCTR-AjCT1-pERK the first four blots.tif]

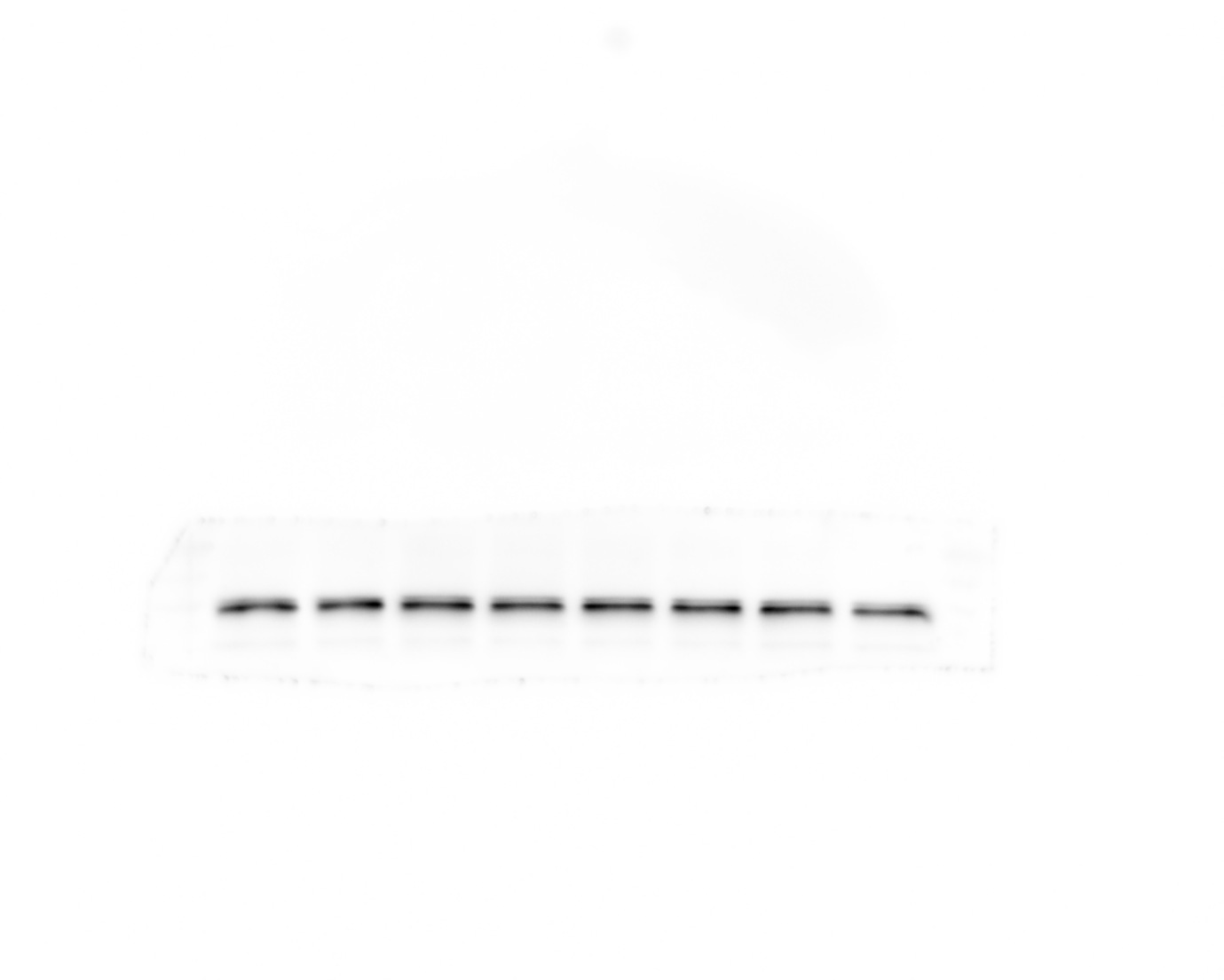

Supplement: Figure 5—figure supplement 1—source data 3. [file elife-101799-fig5-figsupp1-data3.zip › Figure 5-figure supplement 1-1/AjCTR-AjCT1-tERK the last four blots.tif]

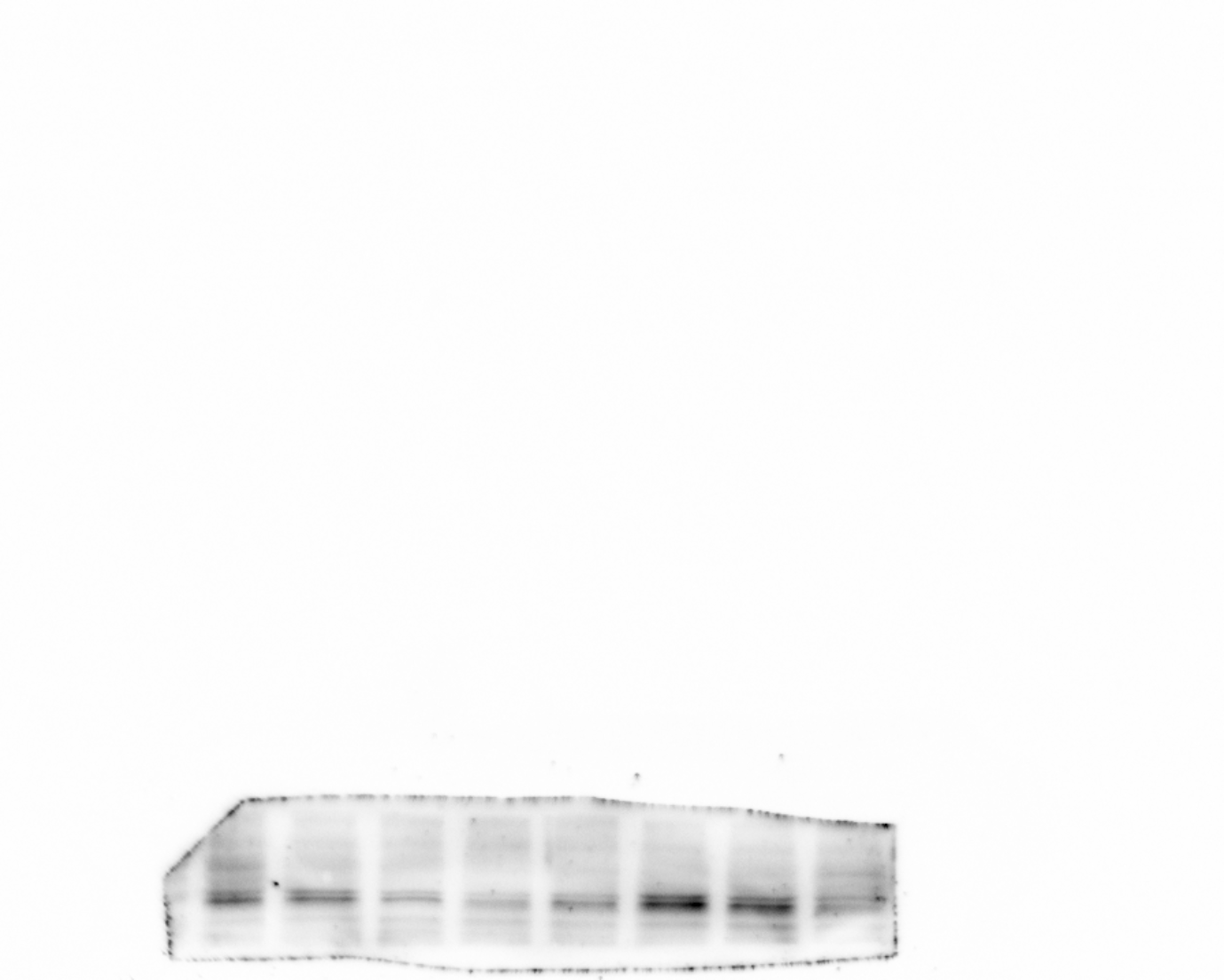

Supplement: Figure 5—figure supplement 1—source data 3. [file elife-101799-fig5-figsupp1-data3.zip › Figure 5-figure supplement 1-1/AjCTR-AjCT2-pERK the last four blots.tif]

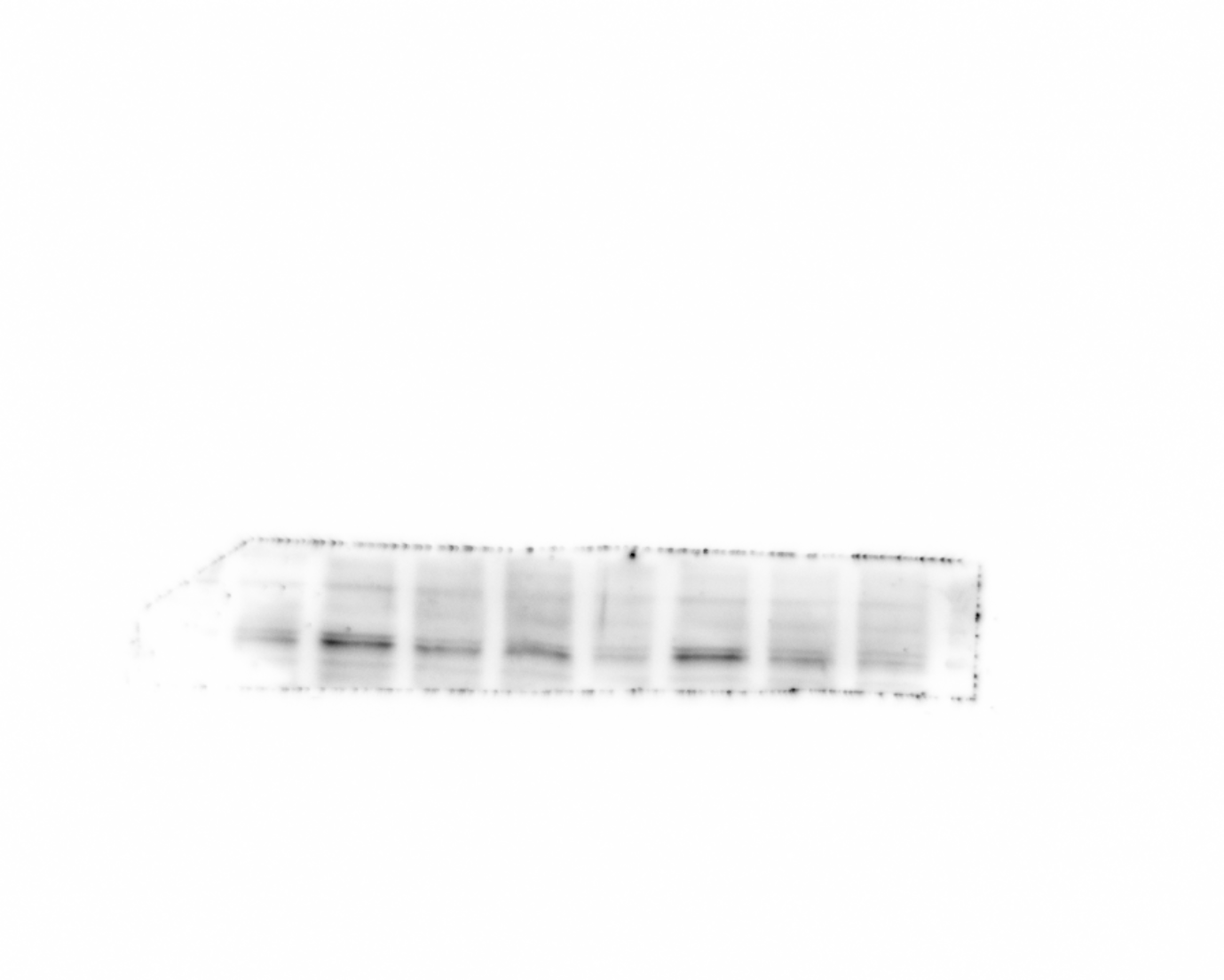

Supplement: Figure 5—figure supplement 1—source data 4. [file elife-101799-fig5-figsupp1-data4.zip › Figure 5-figure supplement 1-2/AjPDFR1-AjCT1-pERK the last four blots.tif]

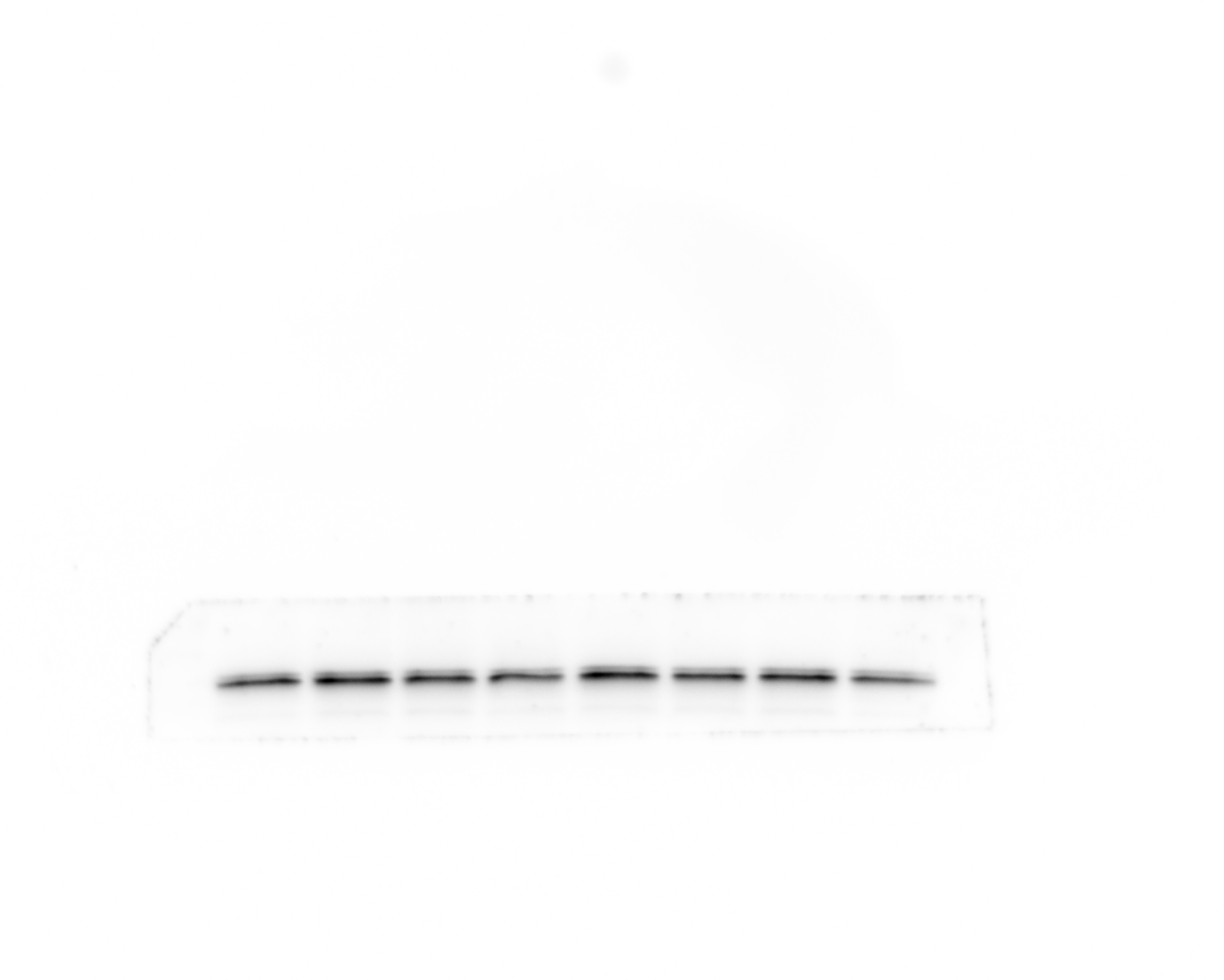

Supplement: Figure 5—figure supplement 1—source data 4. [file elife-101799-fig5-figsupp1-data4.zip › Figure 5-figure supplement 1-2/AjPDFR1-AjCT1-tERK the first four blots.tif]

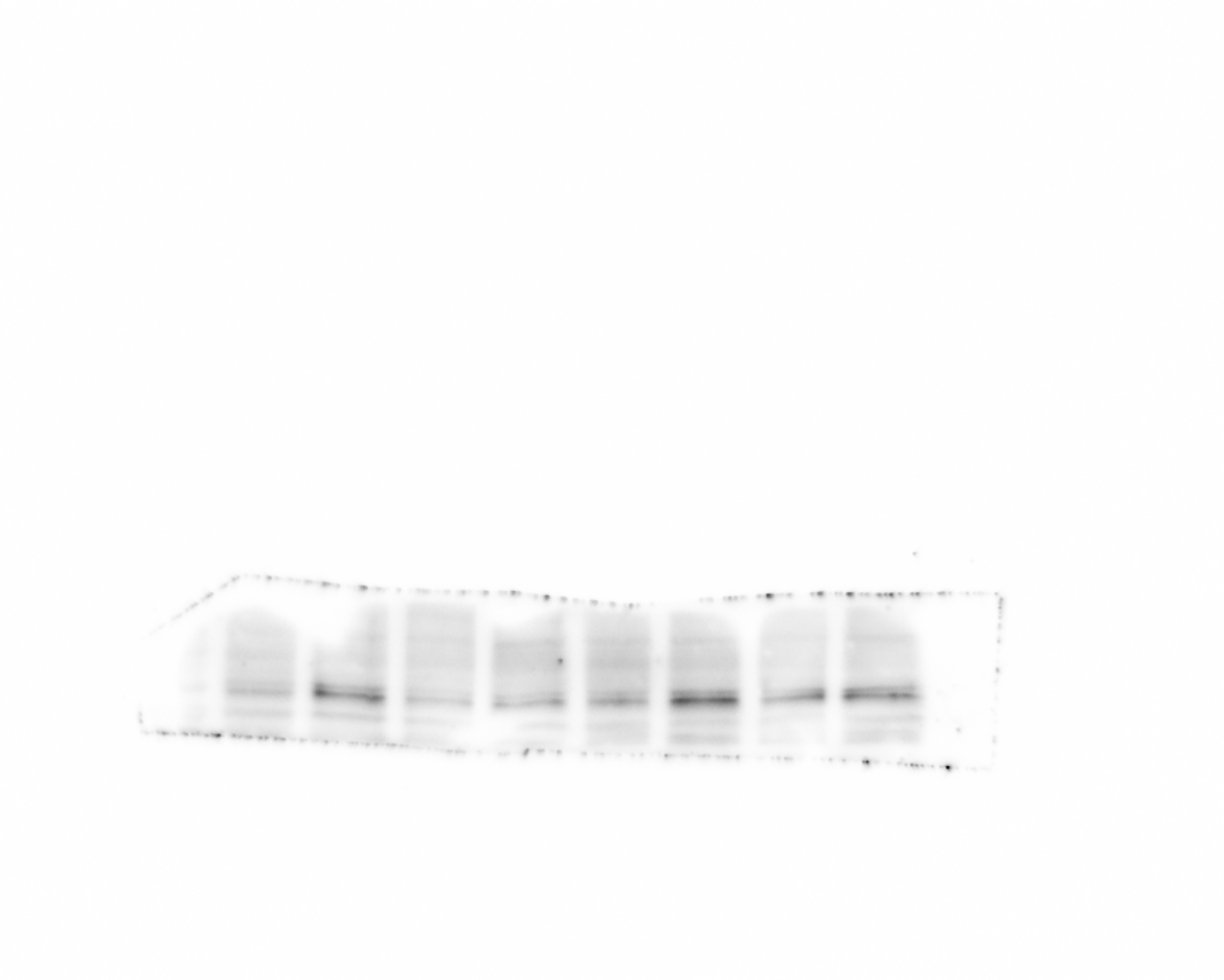

Supplement: Figure 5—figure supplement 1—source data 4. [file elife-101799-fig5-figsupp1-data4.zip › Figure 5-figure supplement 1-2/AjPDFR1-AjCT2-pERK the first four blots.tif]

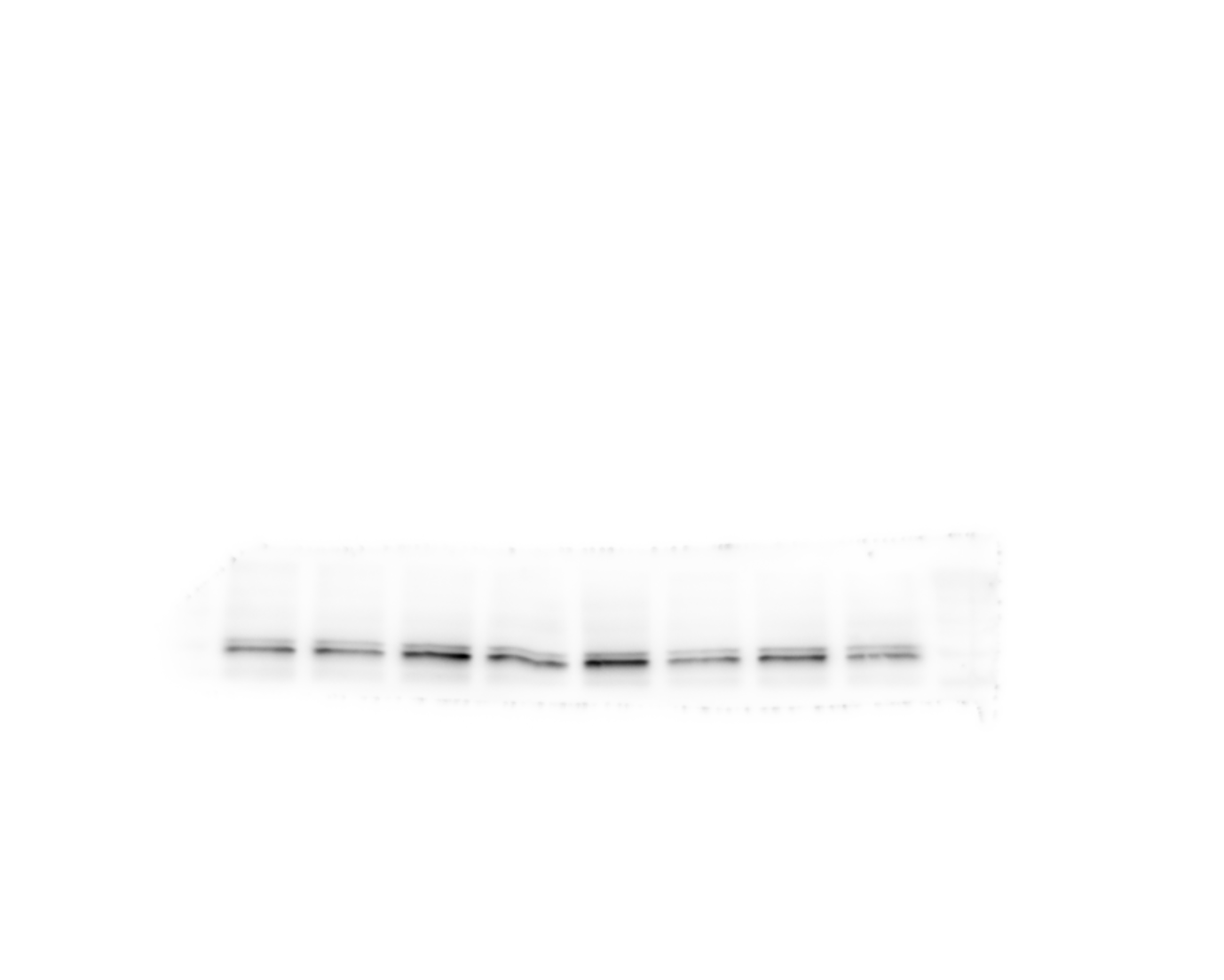

Supplement: Figure 5—figure supplement 1—source data 5. [file elife-101799-fig5-figsupp1-data5.zip › Figure 5-figure supplement 1-3/AjPDFR1-AjCT2-tERK the first four blots.tif]

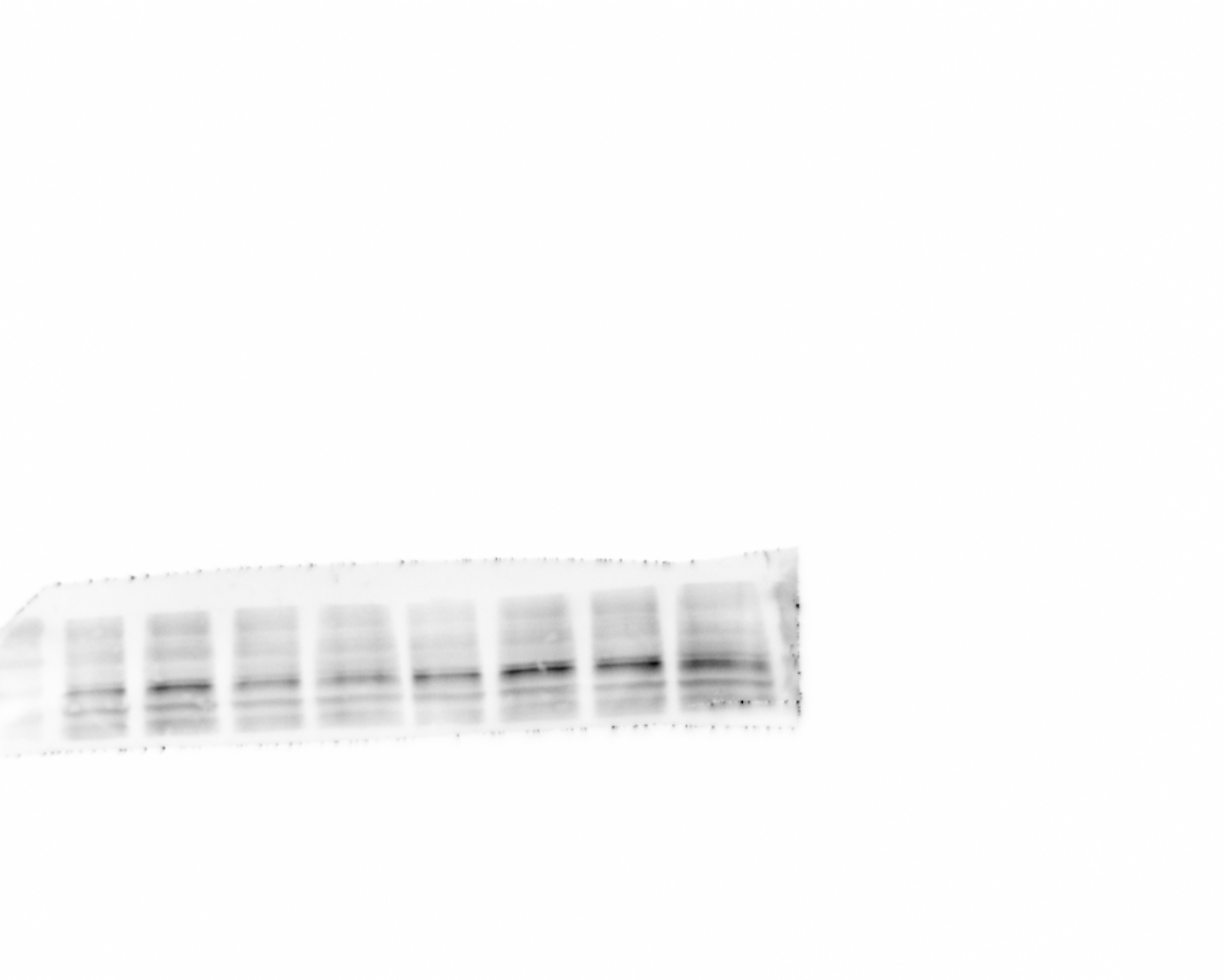

Supplement: Figure 5—figure supplement 1—source data 5. [file elife-101799-fig5-figsupp1-data5.zip › Figure 5-figure supplement 1-3/AjPDFR2-AjCT2-pERK the first four blots.tif]

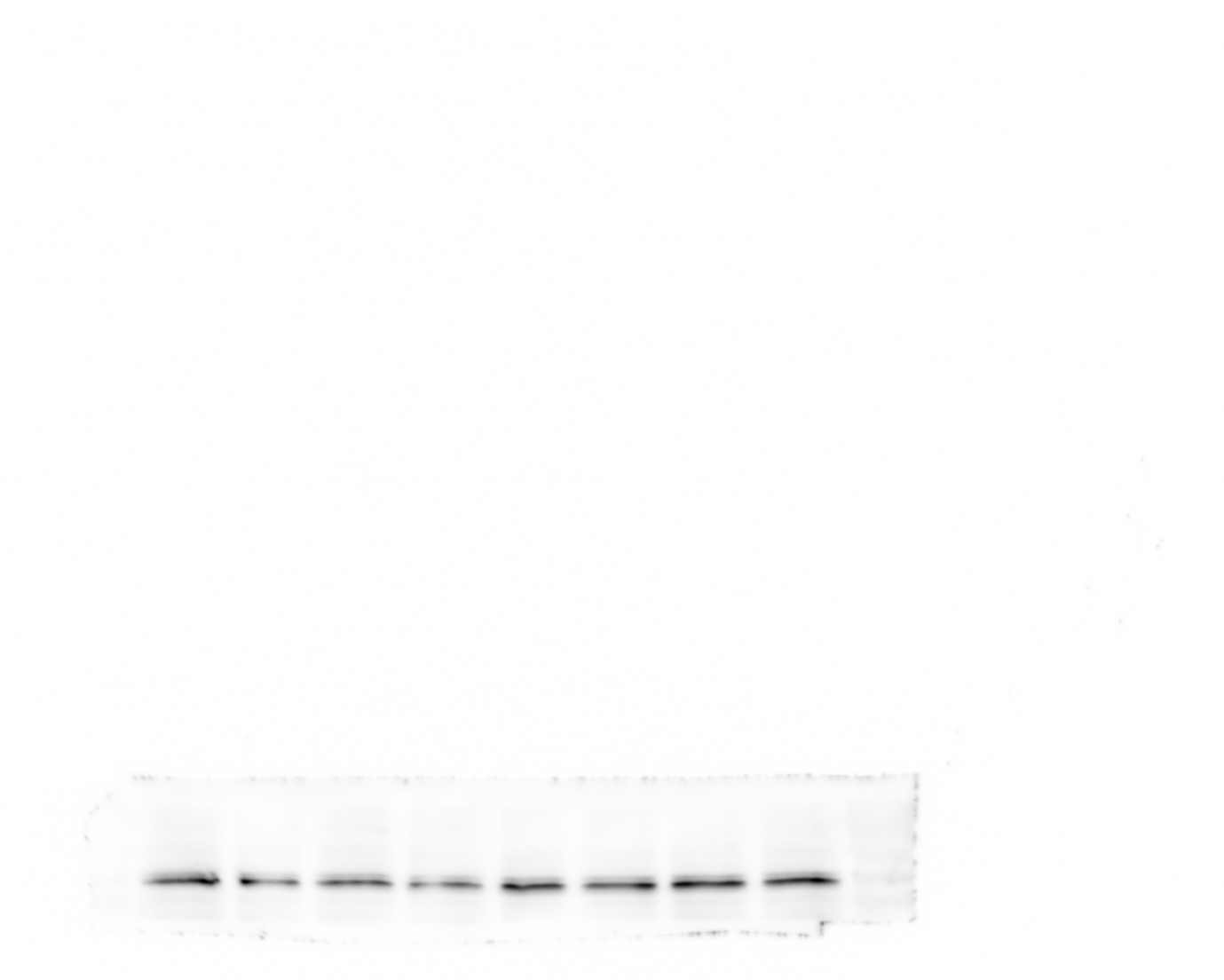

Supplement: Figure 5—figure supplement 1—source data 5. [file elife-101799-fig5-figsupp1-data5.zip › Figure 5-figure supplement 1-3/AjPDFR2-AjCT2-tERK the last four blots.tif]

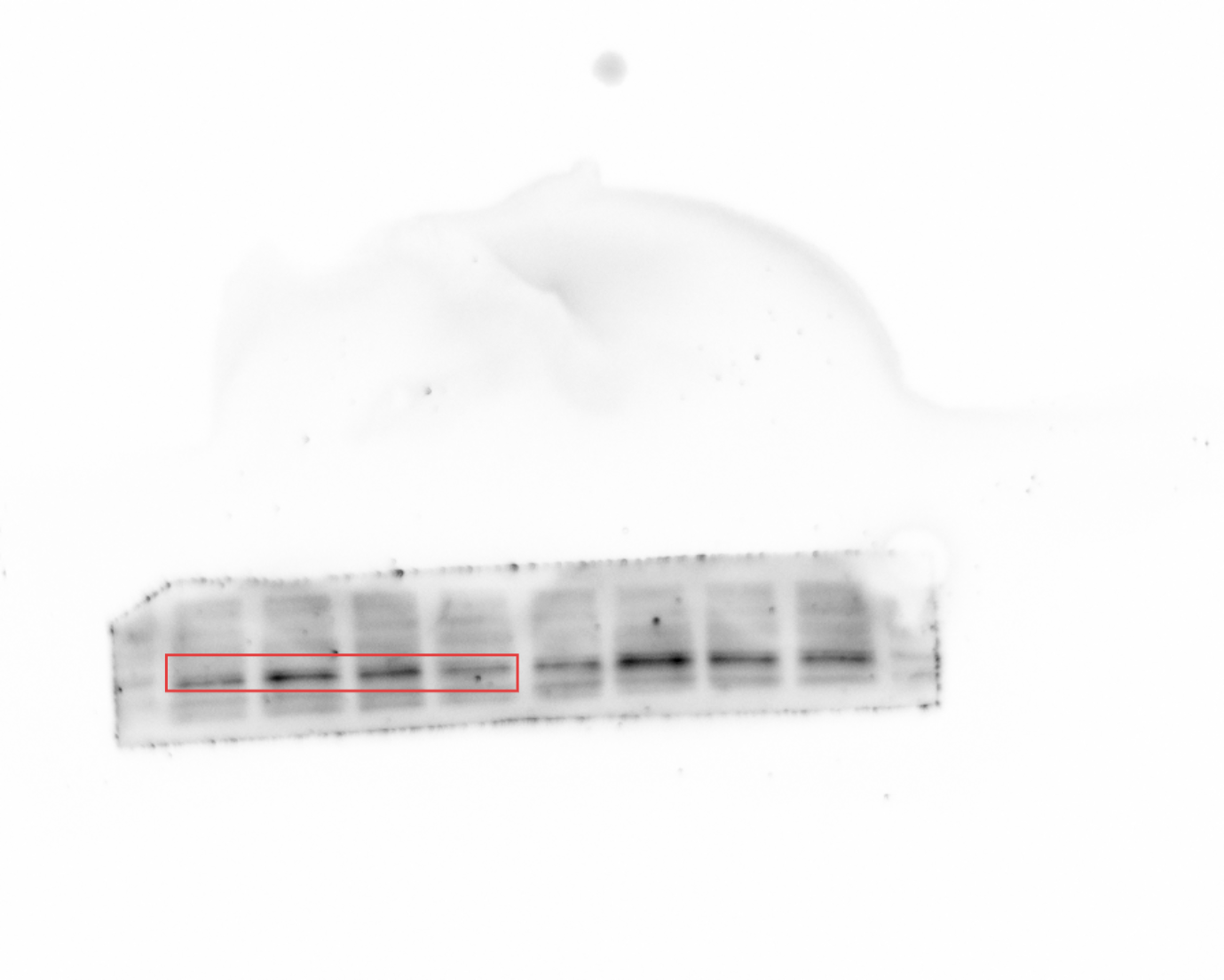

Supplement: Figure 5—figure supplement 1—source data 6. [file elife-101799-fig5-figsupp1-data6.zip › Figure 5-figure supplement 1-Source data3/AjCTR-AjCT1-pERK the first four blots.tif]

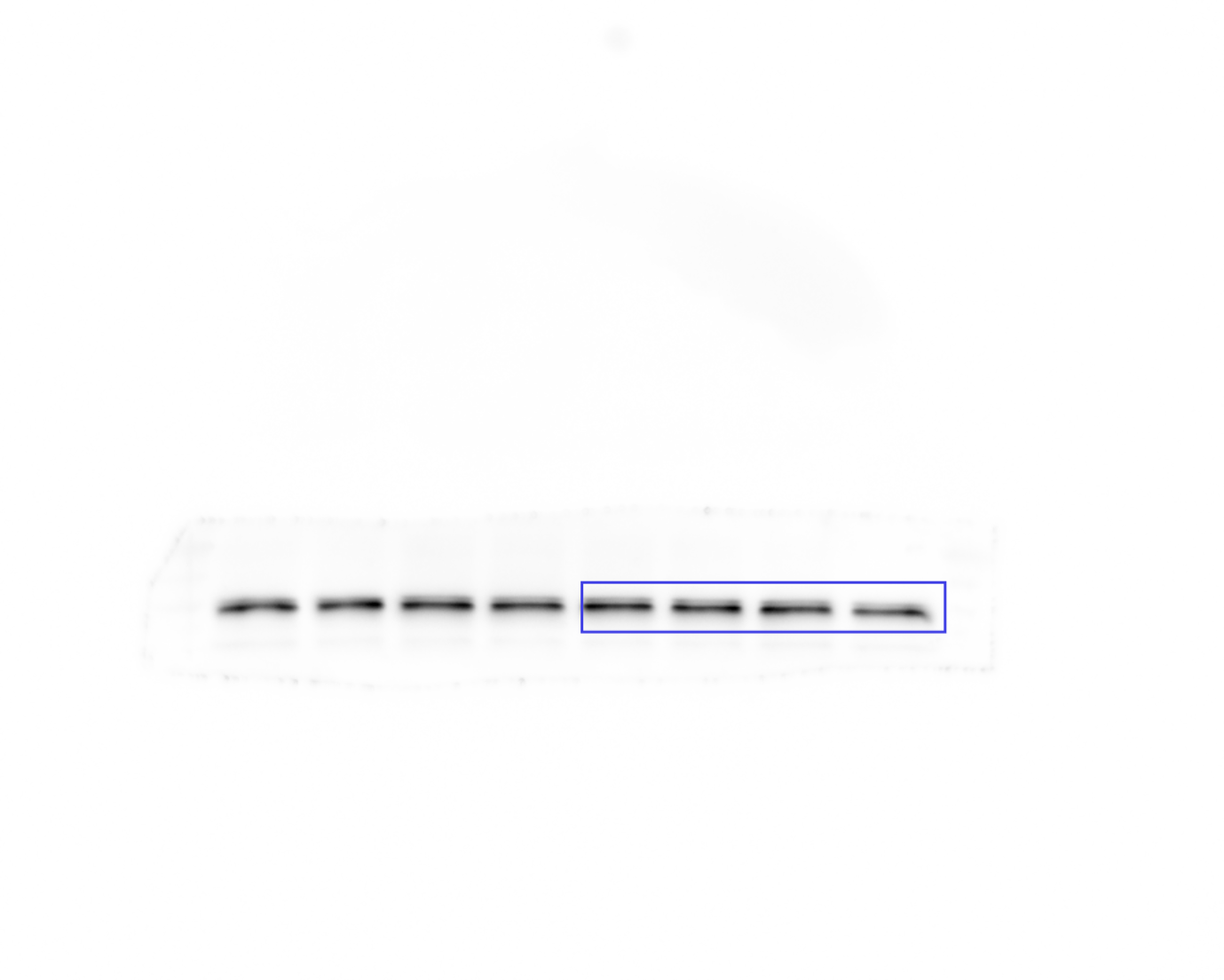

Supplement: Figure 5—figure supplement 1—source data 6. [file elife-101799-fig5-figsupp1-data6.zip › Figure 5-figure supplement 1-Source data3/AjCTR-AjCT1-tERK the last four blots.tif]

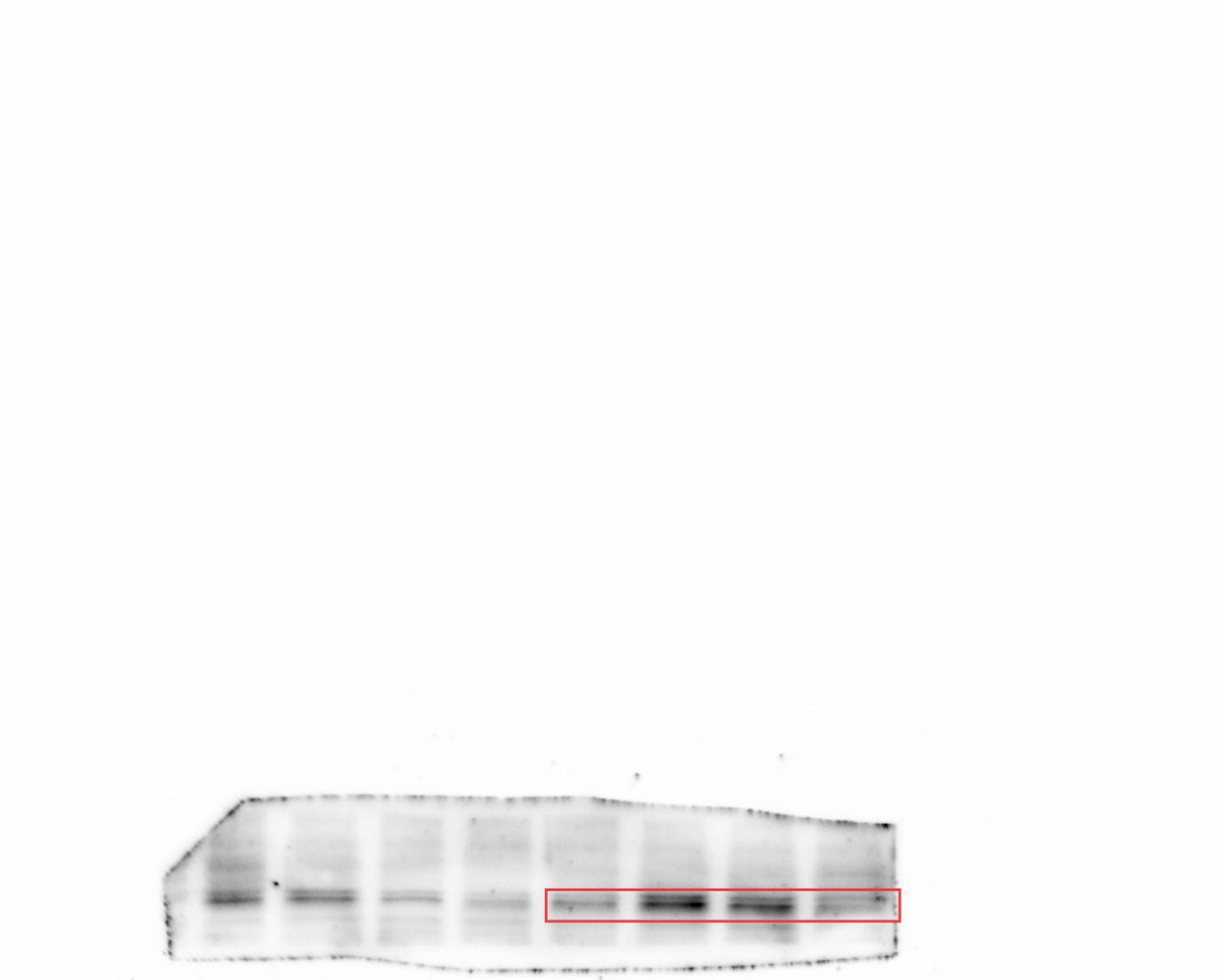

Supplement: Figure 5—figure supplement 1—source data 6. [file elife-101799-fig5-figsupp1-data6.zip › Figure 5-figure supplement 1-Source data3/AjCTR-AjCT2-pERK the last four blots.tif]

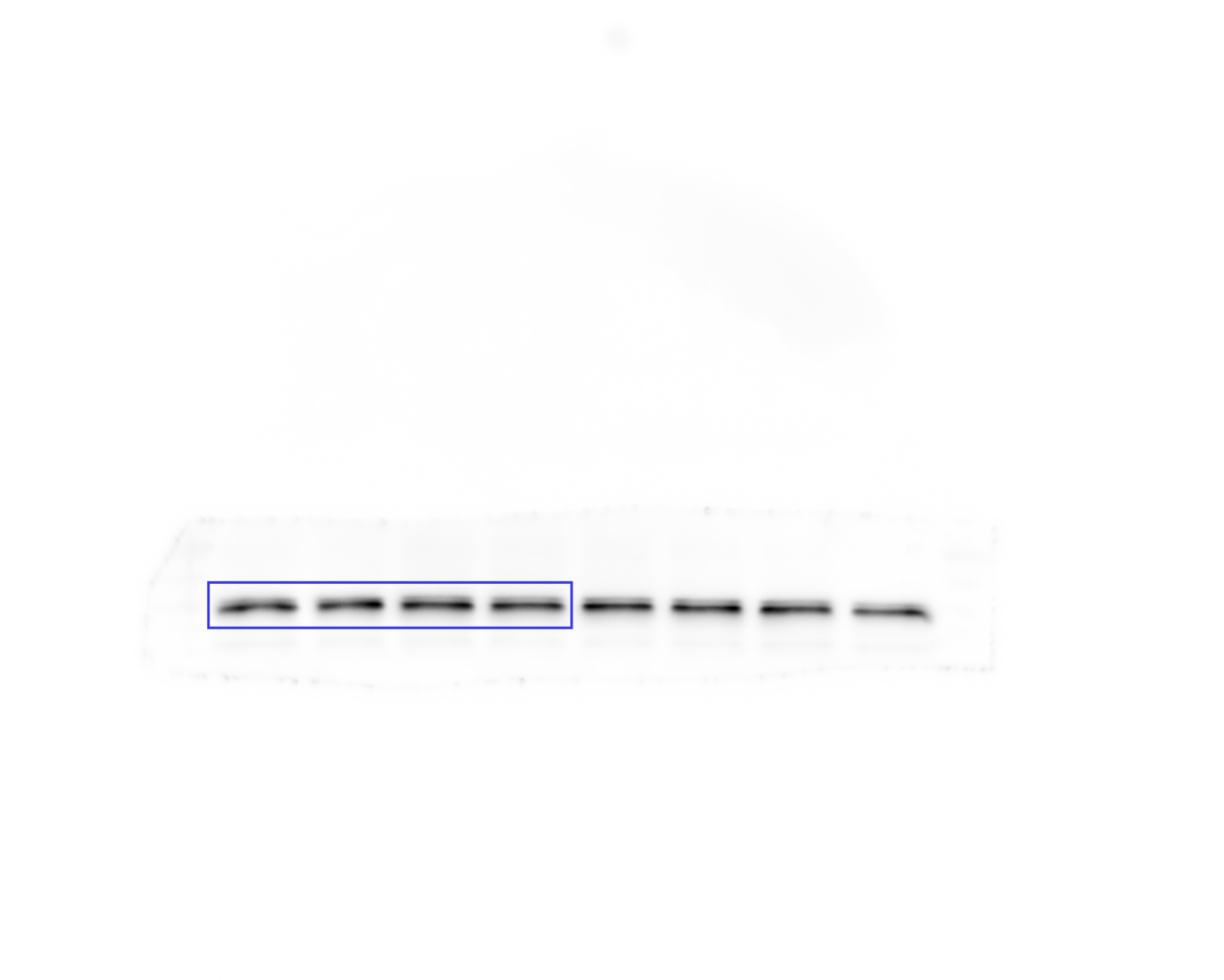

Supplement: Figure 5—figure supplement 1—source data 6. [file elife-101799-fig5-figsupp1-data6.zip › Figure 5-figure supplement 1-Source data3/AjCTR-AjCT2-tERK the first four blots.tif]

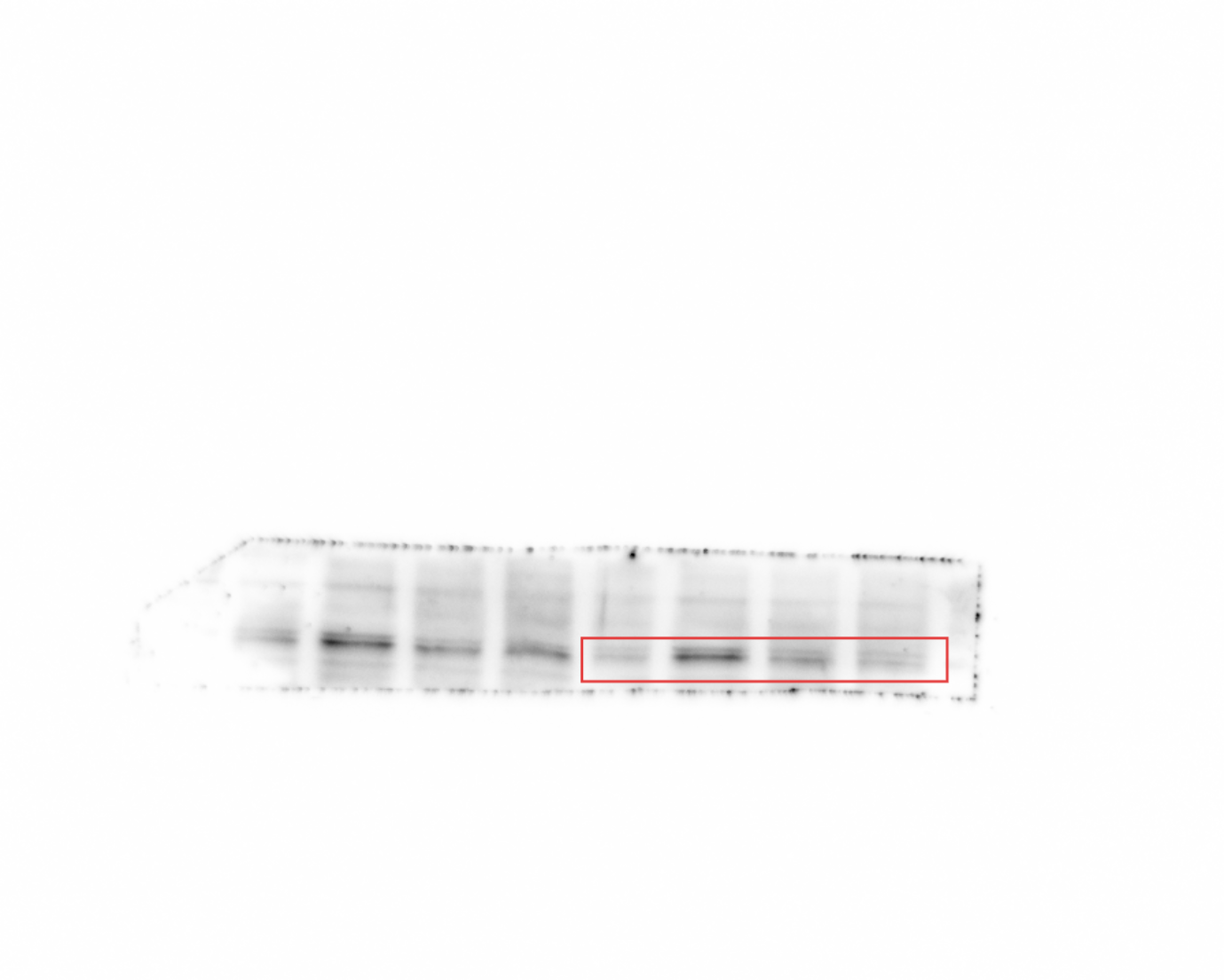

Supplement: Figure 5—figure supplement 1—source data 6. [file elife-101799-fig5-figsupp1-data6.zip › Figure 5-figure supplement 1-Source data3/AjPDFR1-AjCT1-pERK the last four blots.tif]

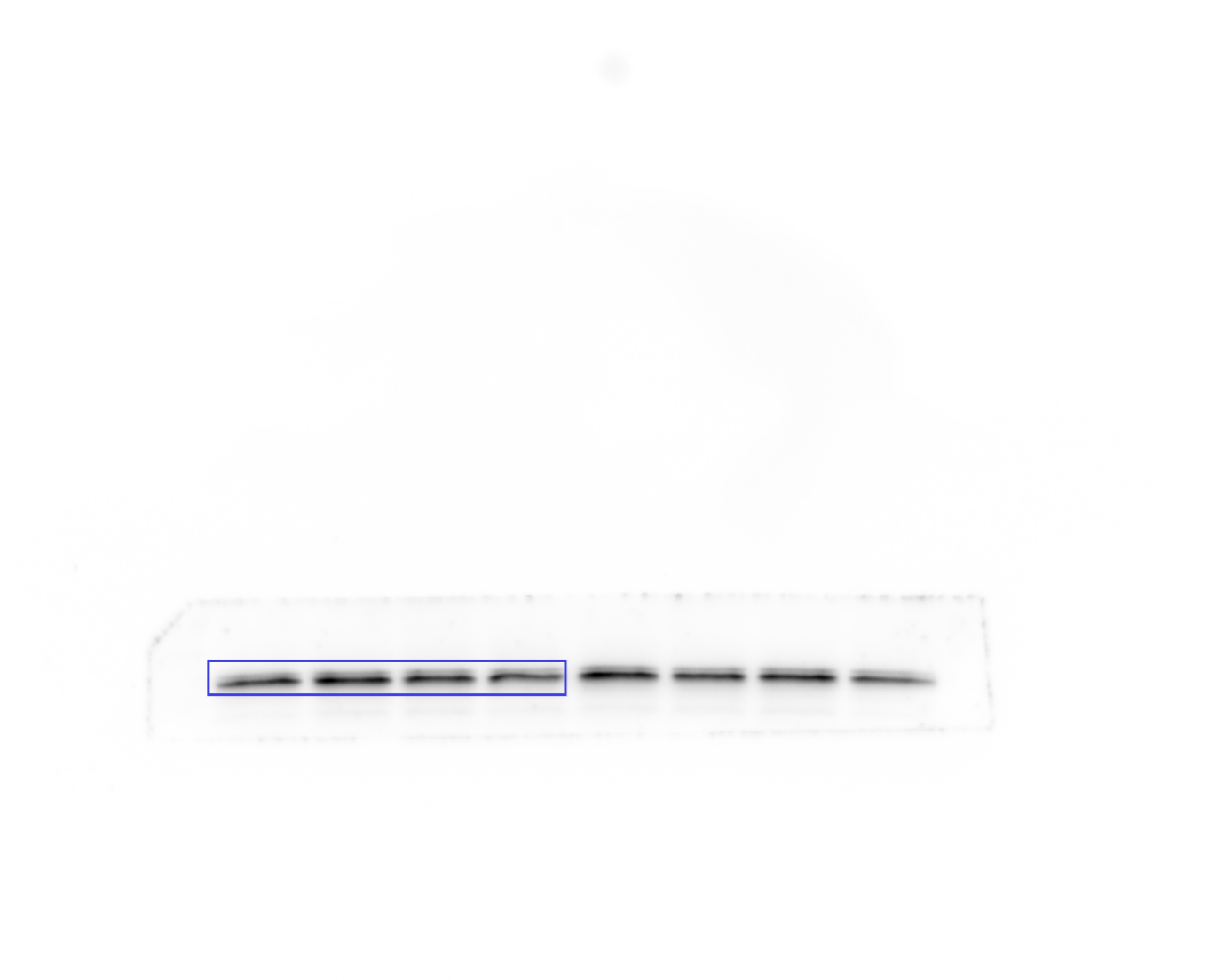

Supplement: Figure 5—figure supplement 1—source data 6. [file elife-101799-fig5-figsupp1-data6.zip › Figure 5-figure supplement 1-Source data3/AjPDFR1-AjCT1-tERK the first four blots.tif]

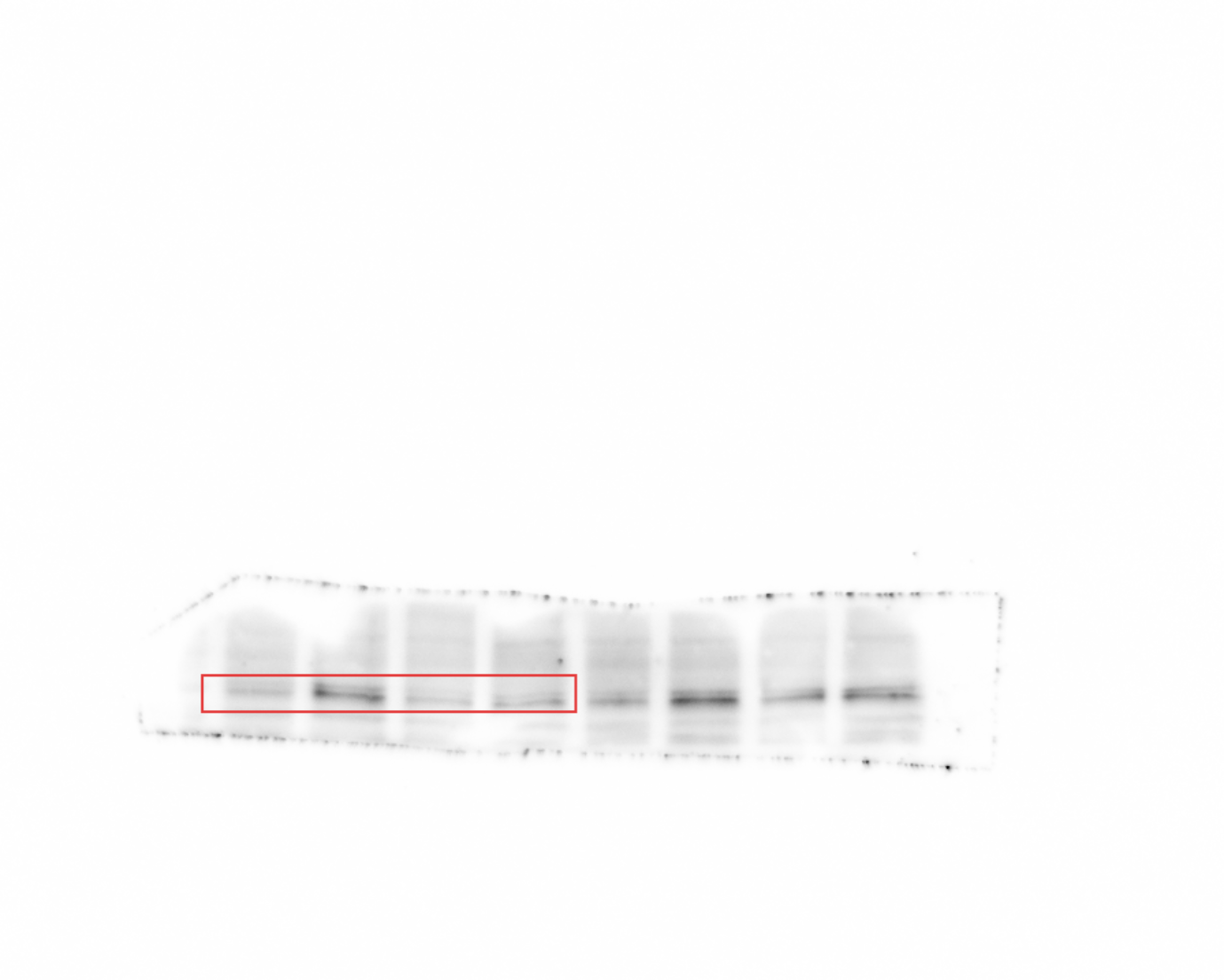

Supplement: Figure 5—figure supplement 1—source data 6. [file elife-101799-fig5-figsupp1-data6.zip › Figure 5-figure supplement 1-Source data3/AjPDFR1-AjCT2-pERK the first four blots.tif]

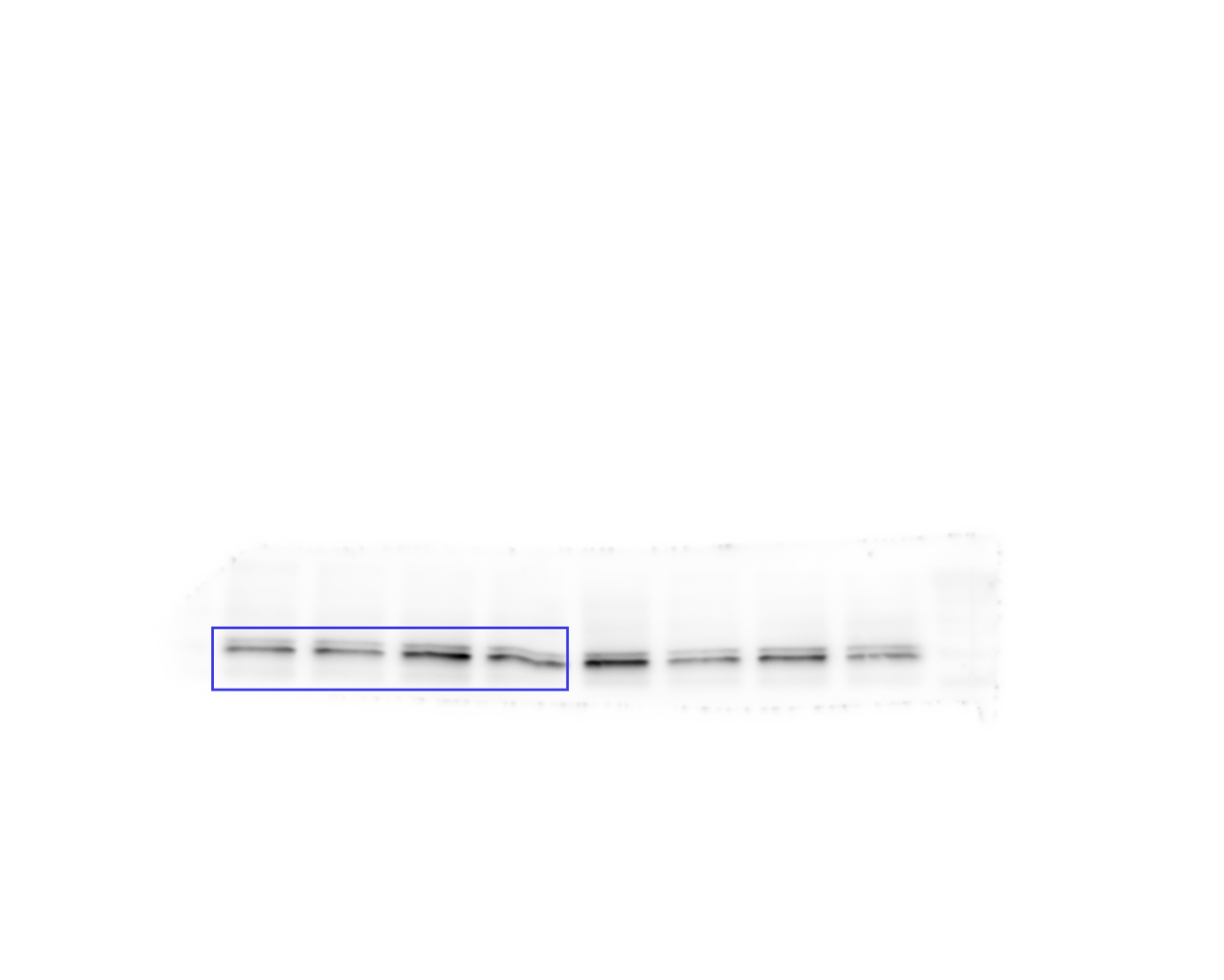

Supplement: Figure 5—figure supplement 1—source data 6. [file elife-101799-fig5-figsupp1-data6.zip › Figure 5-figure supplement 1-Source data3/AjPDFR1-AjCT2-tERK the first four blots.tif]

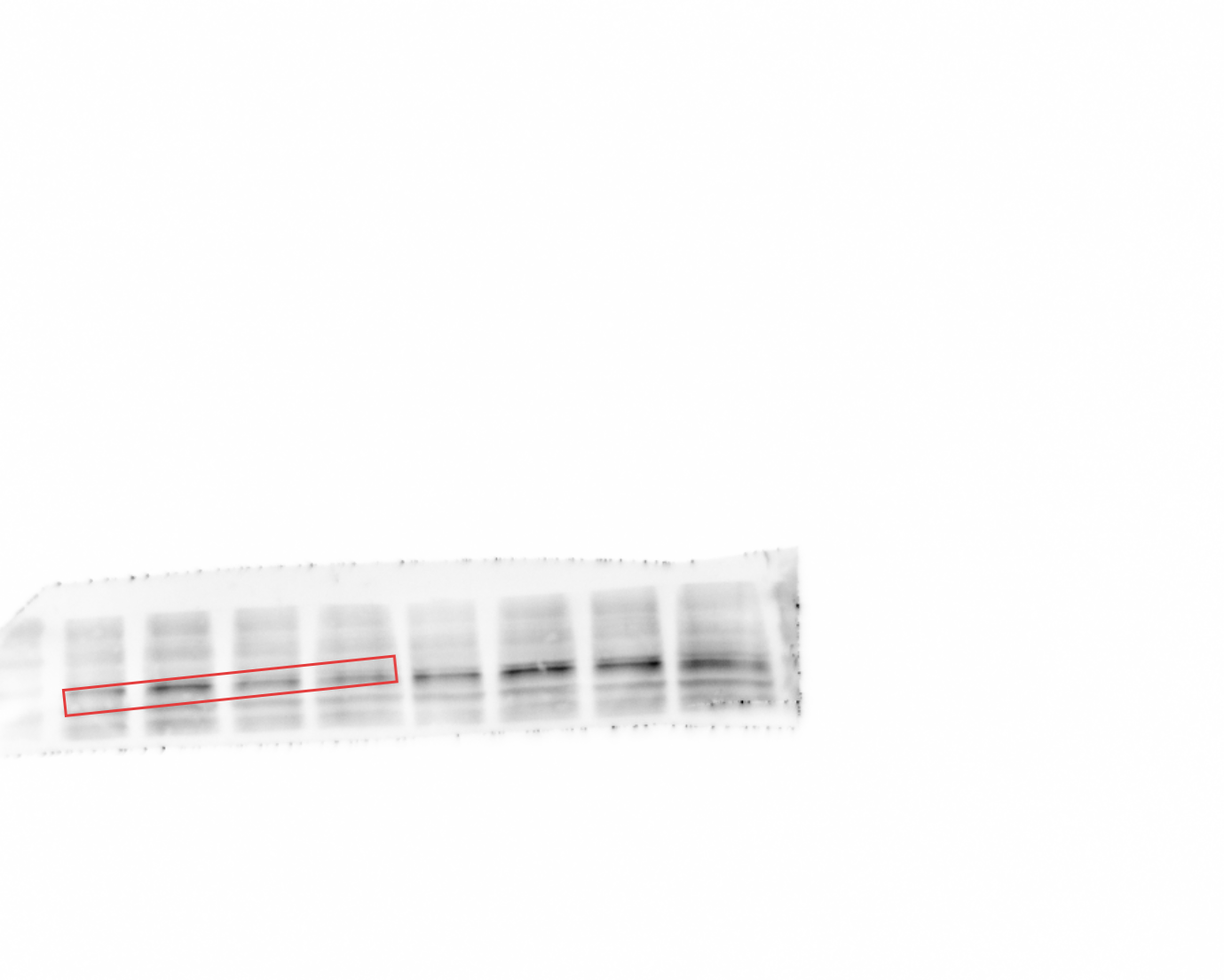

Supplement: Figure 5—figure supplement 1—source data 6. [file elife-101799-fig5-figsupp1-data6.zip › Figure 5-figure supplement 1-Source data3/AjPDFR2-AjCT2-pERK the first four blots.tif]

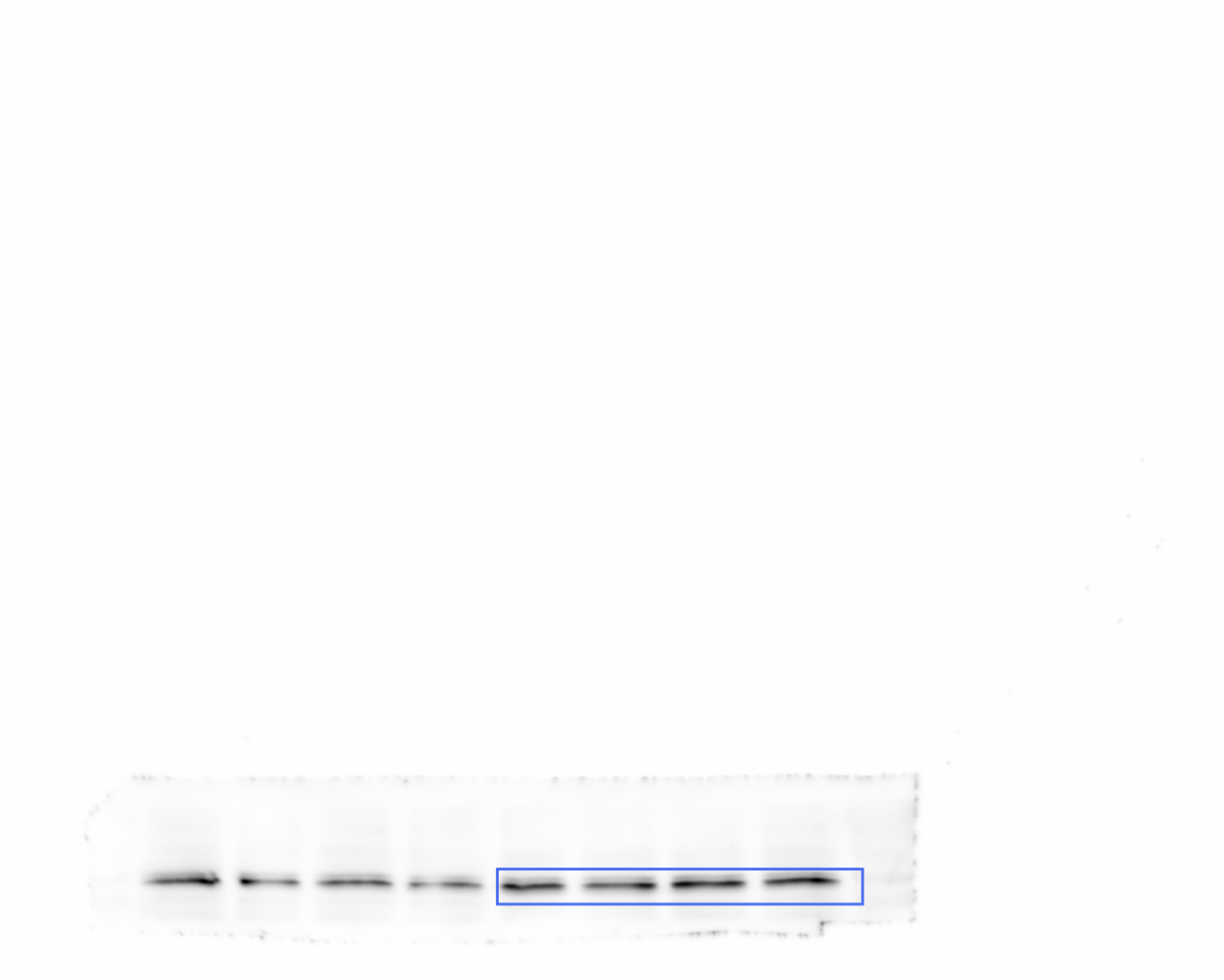

Supplement: Figure 5—figure supplement 1—source data 6. [file elife-101799-fig5-figsupp1-data6.zip › Figure 5-figure supplement 1-Source data3/AjPDFR2-AjCT2-tERK the last four blots.tif]

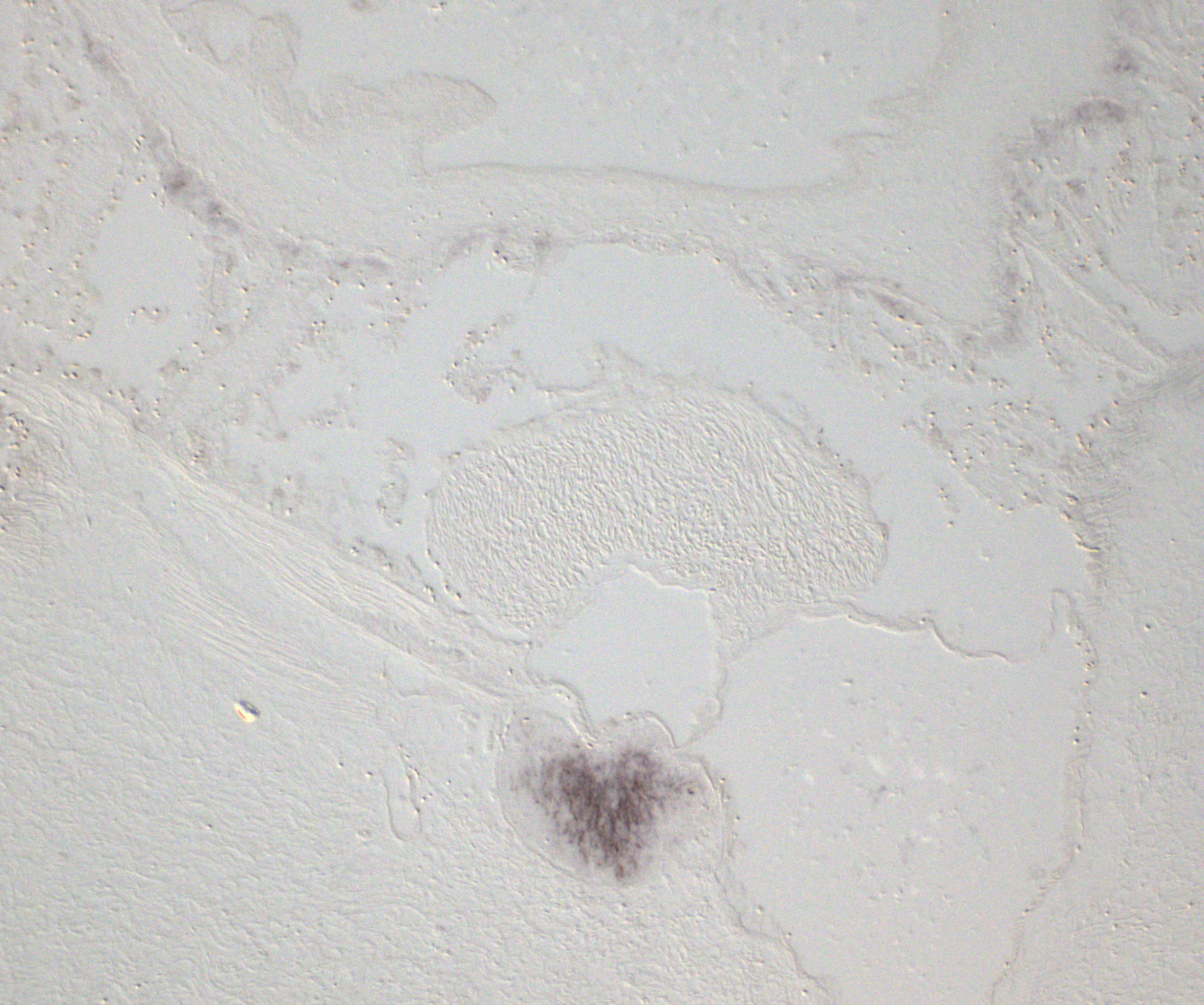

Supplement: Figure 7—source data 1. — 2.5 AjCT1 and AjCT2 cause dose-dependent relaxation of longitudinal muscle and intestine preparations from A. japonicus. [file elife-101799-fig7-data1.zip › Figure 7-Source data1/Figure 7A.tif]

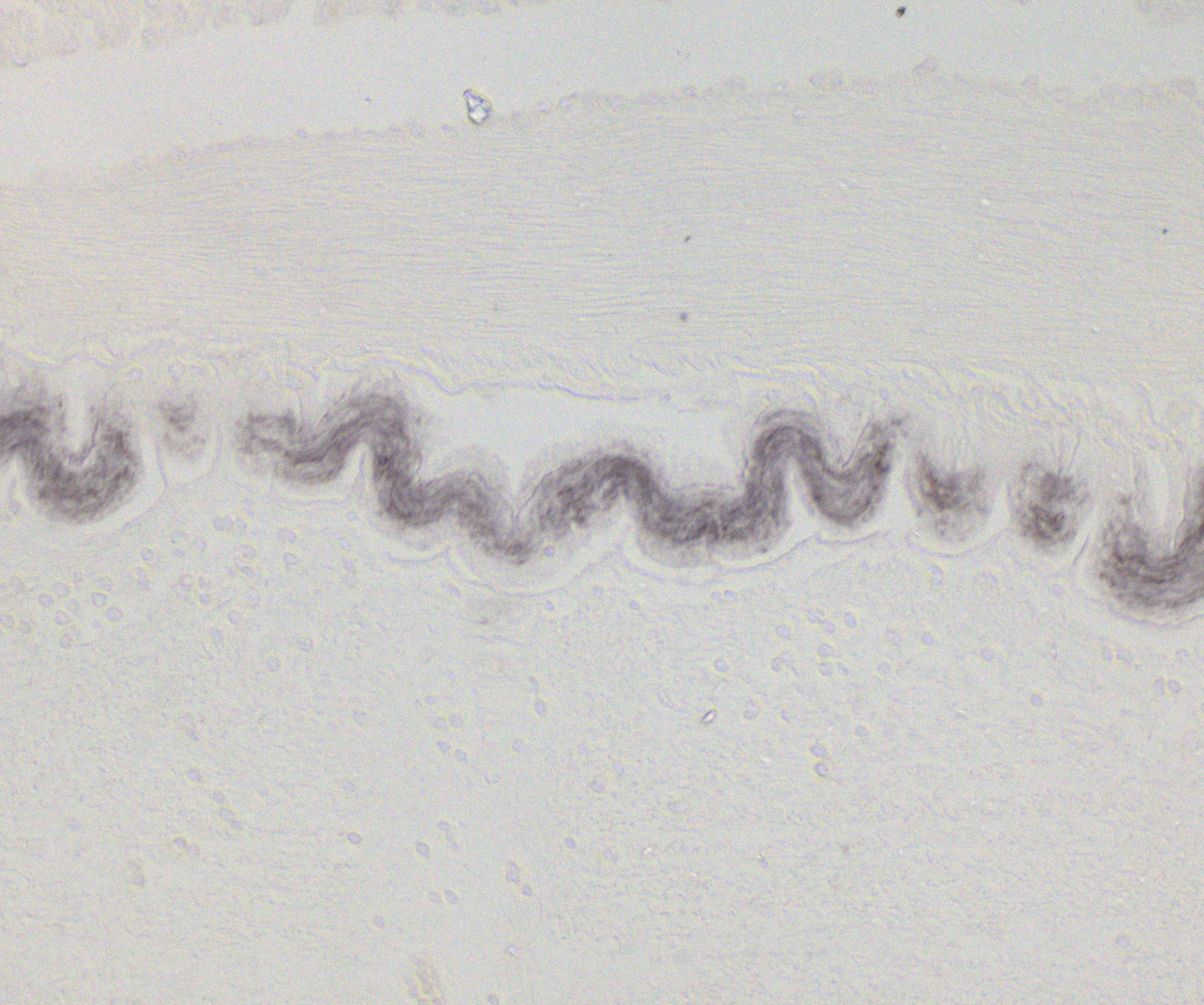

Supplement: Figure 7—source data 1. — 2.5 AjCT1 and AjCT2 cause dose-dependent relaxation of longitudinal muscle and intestine preparations from A. japonicus. [file elife-101799-fig7-data1.zip › Figure 7-Source data1/Figure 7B.tif]

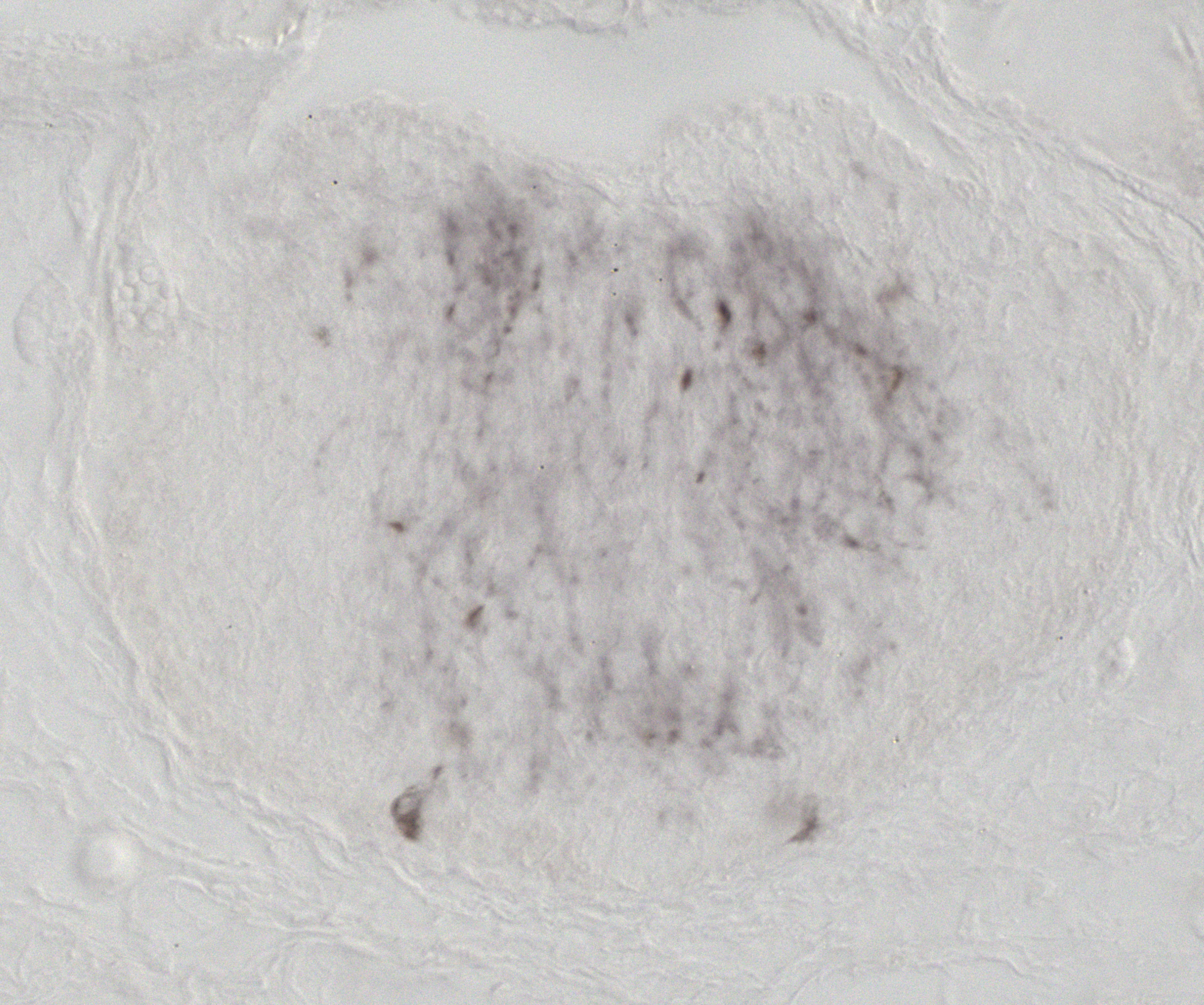

Supplement: Figure 7—source data 1. — 2.5 AjCT1 and AjCT2 cause dose-dependent relaxation of longitudinal muscle and intestine preparations from A. japonicus. [file elife-101799-fig7-data1.zip › Figure 7-Source data1/Figure 7C.tif]

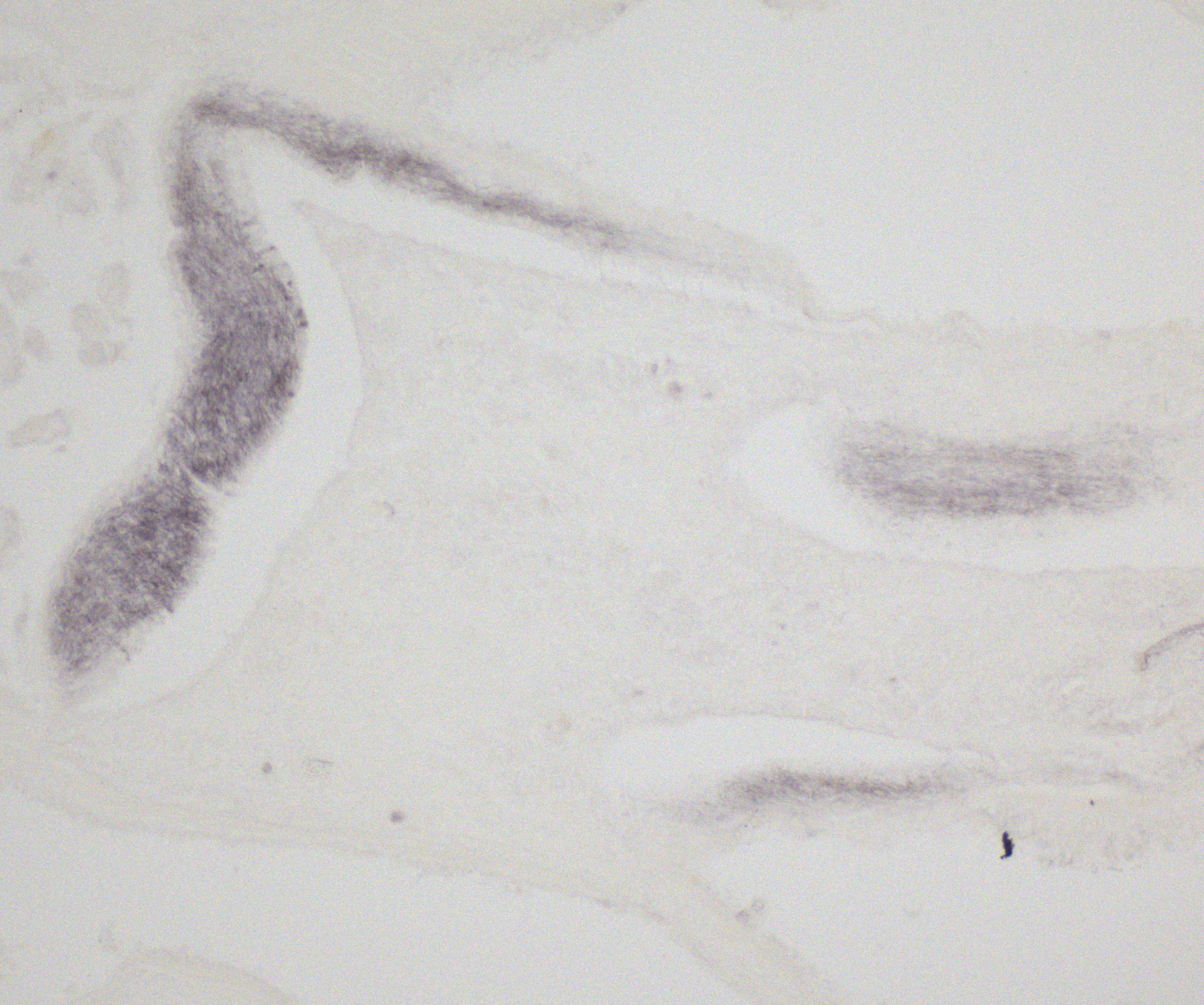

Supplement: Figure 7—source data 1. — 2.5 AjCT1 and AjCT2 cause dose-dependent relaxation of longitudinal muscle and intestine preparations from A. japonicus. [file elife-101799-fig7-data1.zip › Figure 7-Source data1/Figure 7D.tif]

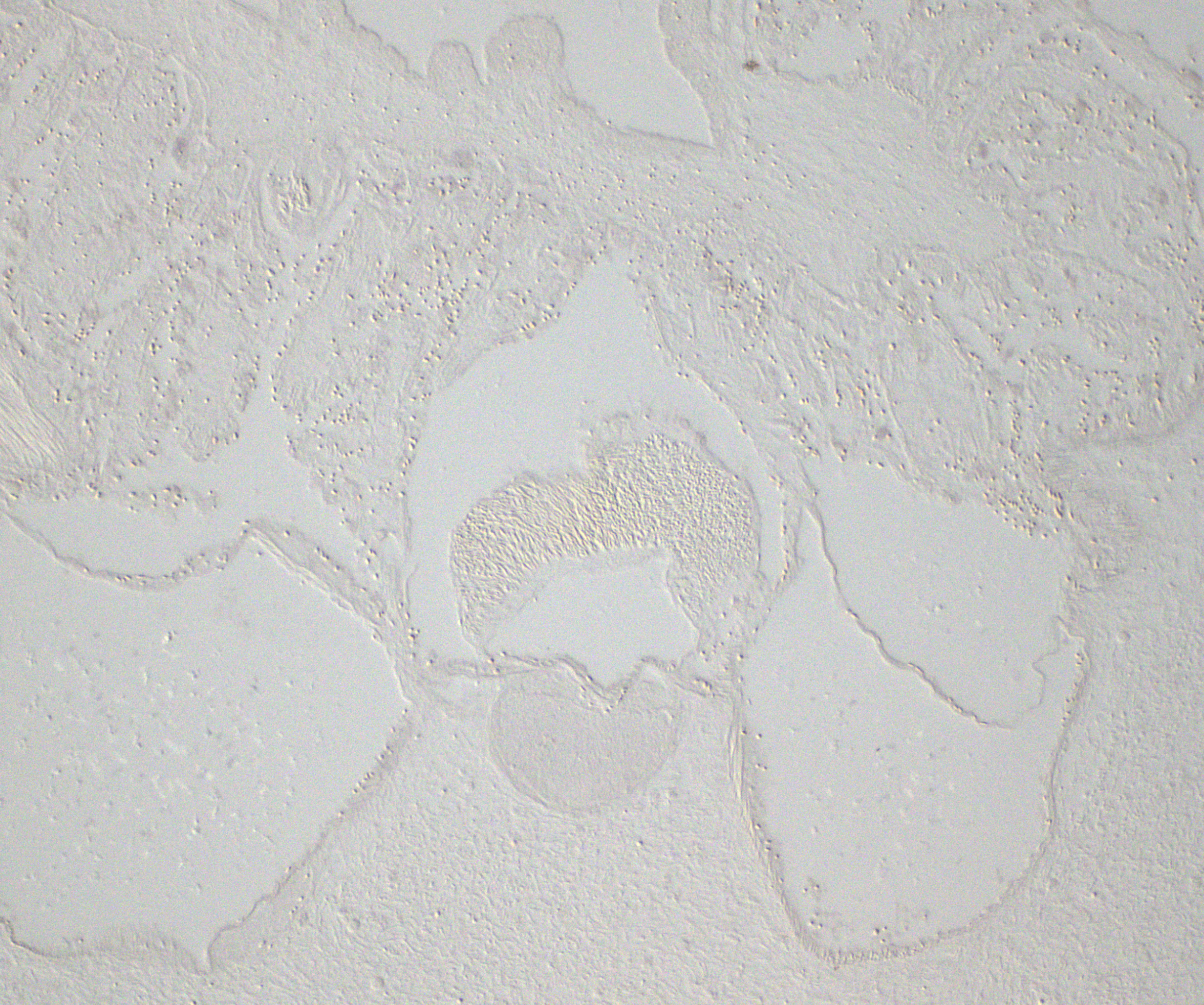

Supplement: Figure 7—figure supplement 1—source data 1. [file elife-101799-fig7-figsupp1-data1.zip › Figure 7-figure supplement 1/Figure 7-figure supplement 1B.tif]

**A**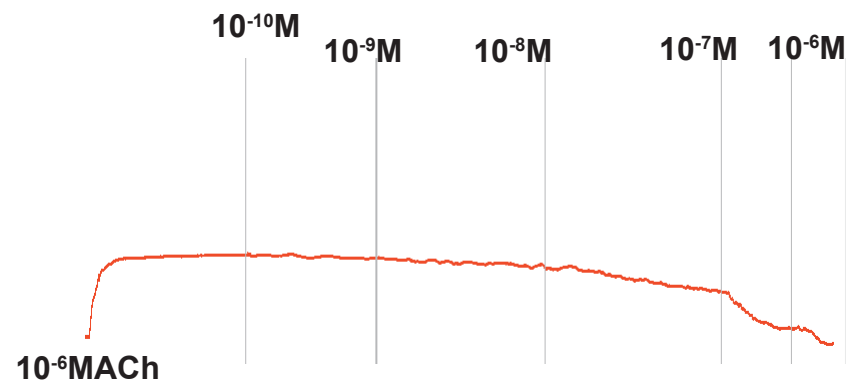**B**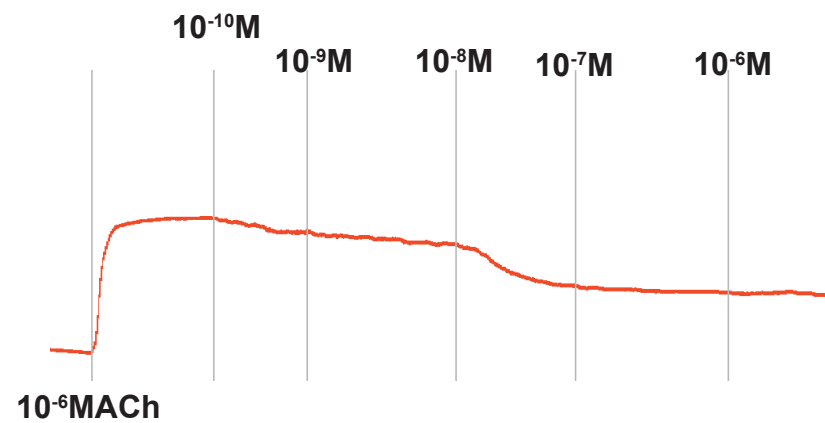**C**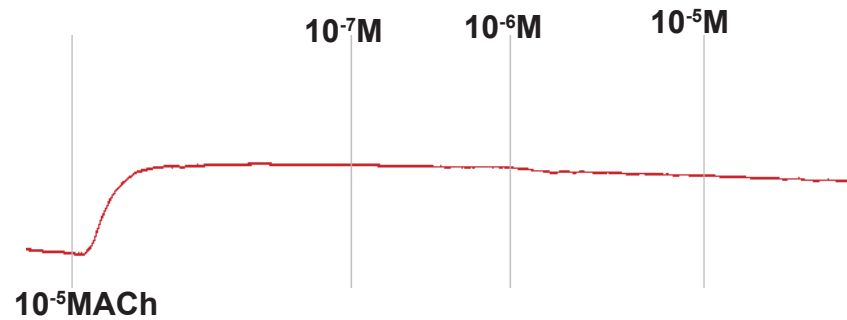**D**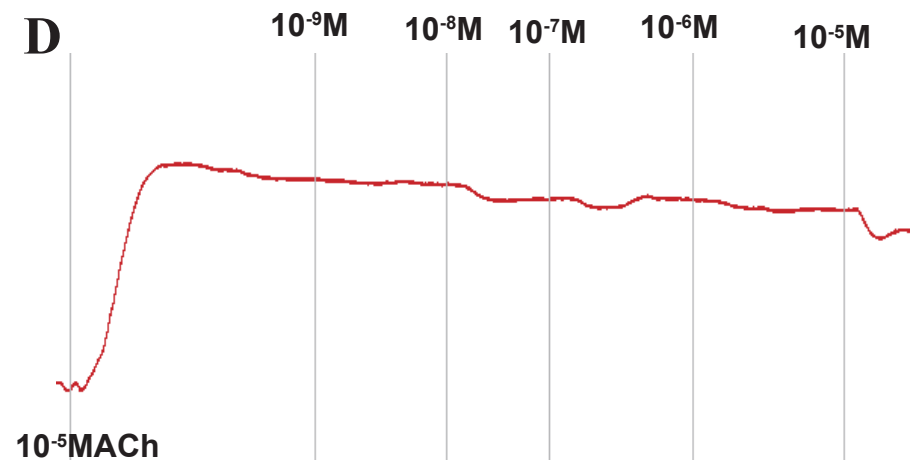

Supplement: Figure 8—source data 1. [file elife-101799-fig8-data1.zip › Figure 8-Source data/Figure 8-Source data1.pdf]

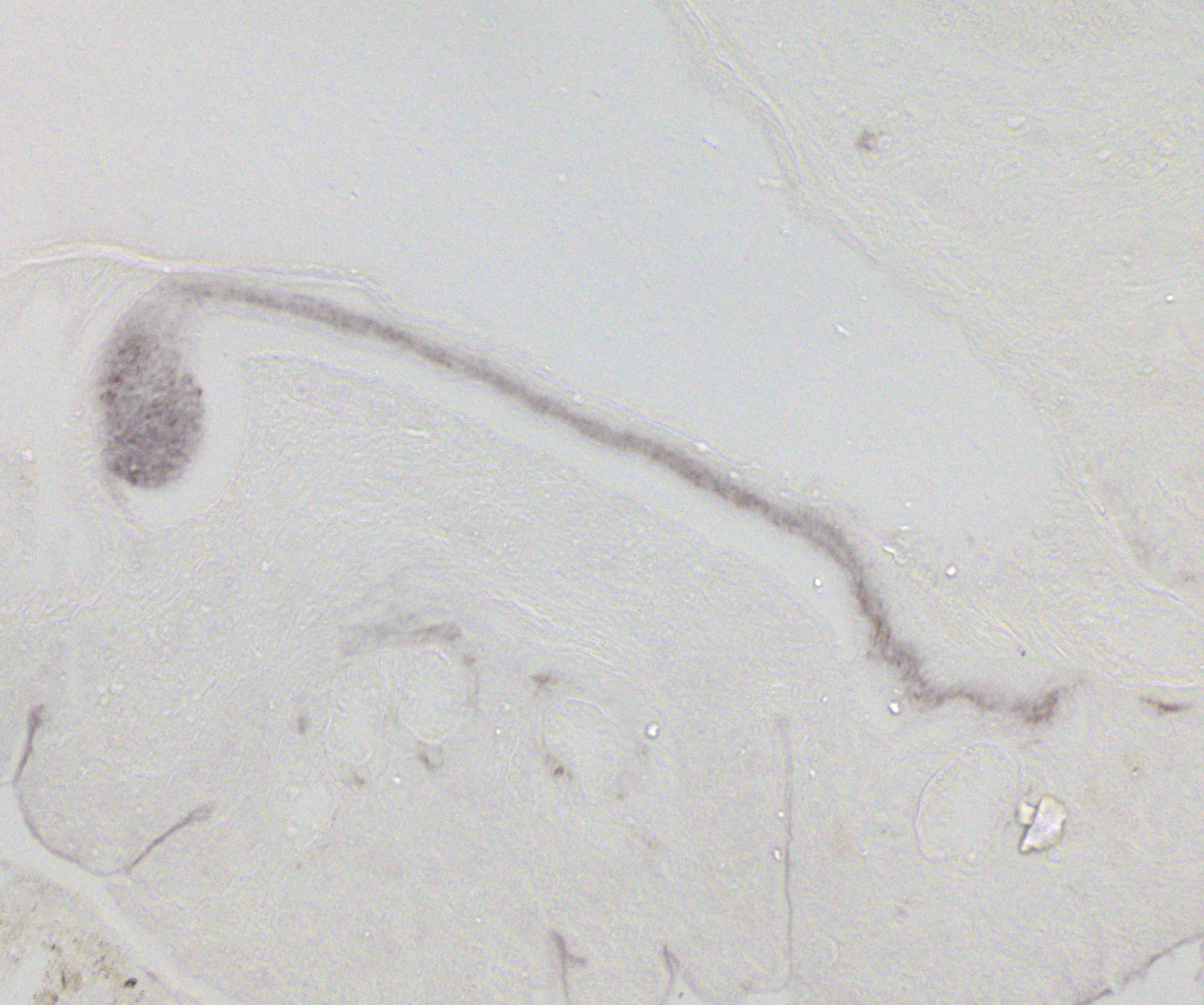

Supplement: Figure 8—source data 2. [file elife-101799-fig8-data2.zip › Figure 7-Source data 2/Figure 7E.tif]

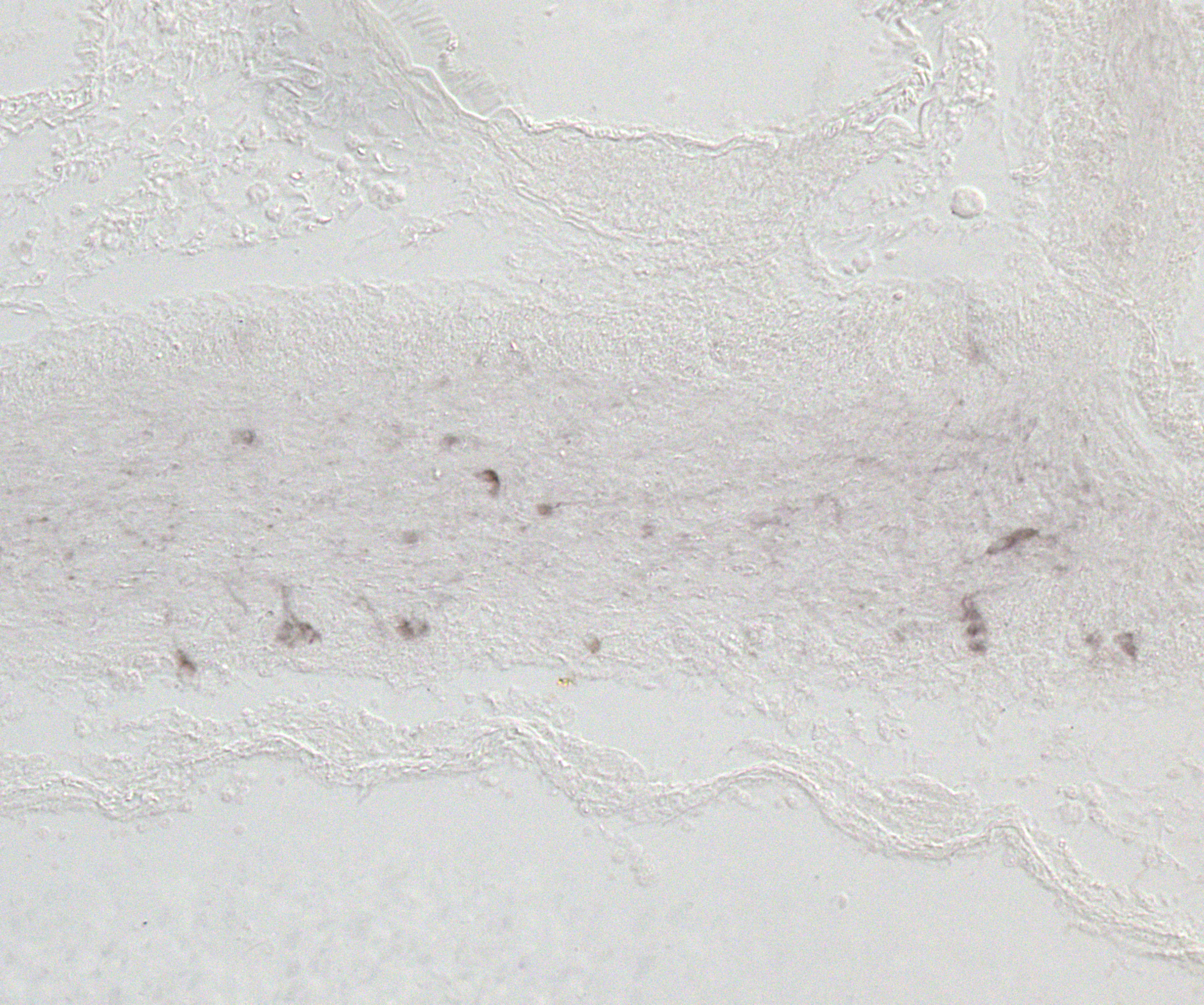

Supplement: Figure 8—source data 2. [file elife-101799-fig8-data2.zip › Figure 7-Source data 2/Figure 7F.tif]

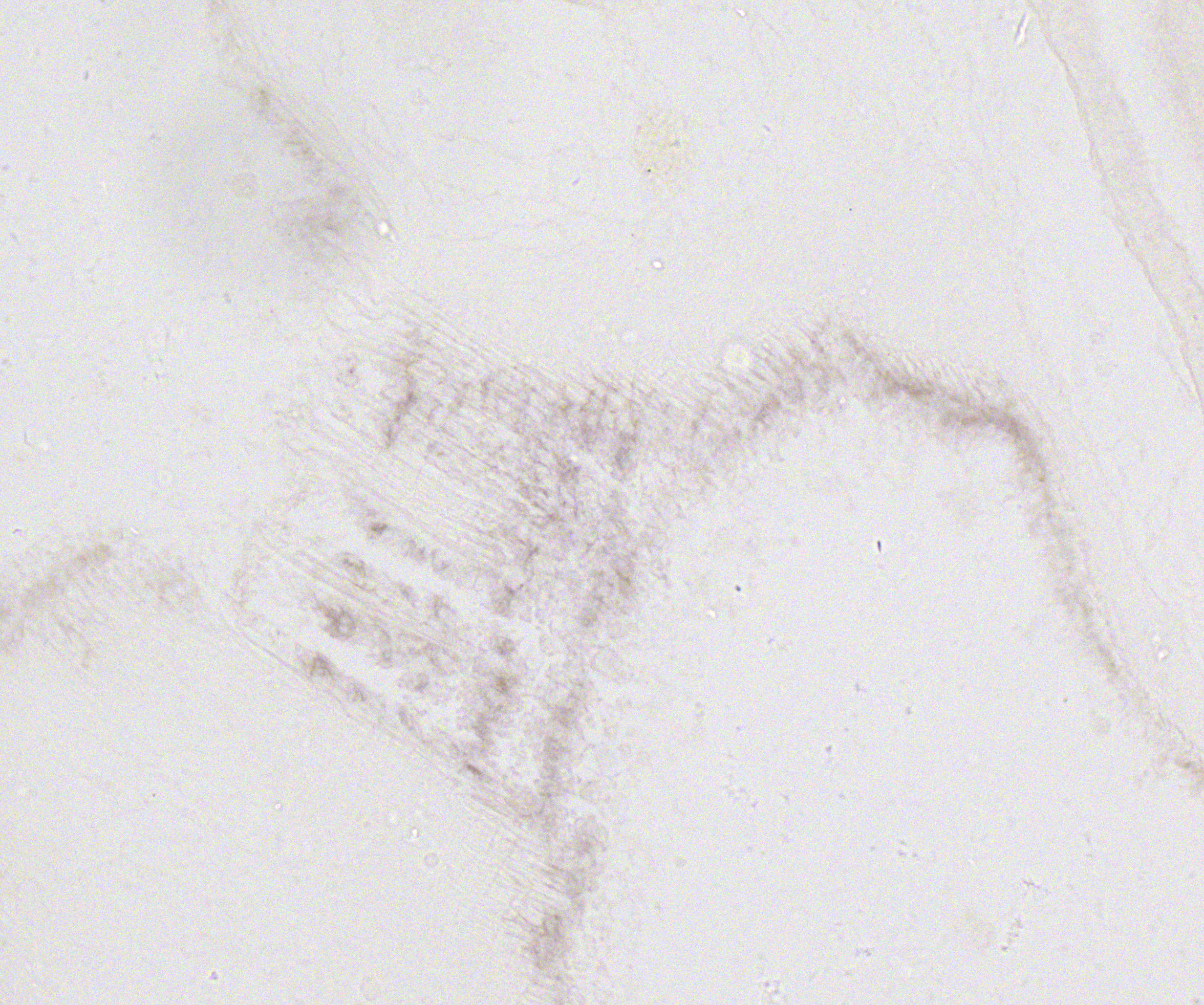

Supplement: Figure 8—source data 2. [file elife-101799-fig8-data2.zip › Figure 7-Source data 2/Figure 7G.tif]

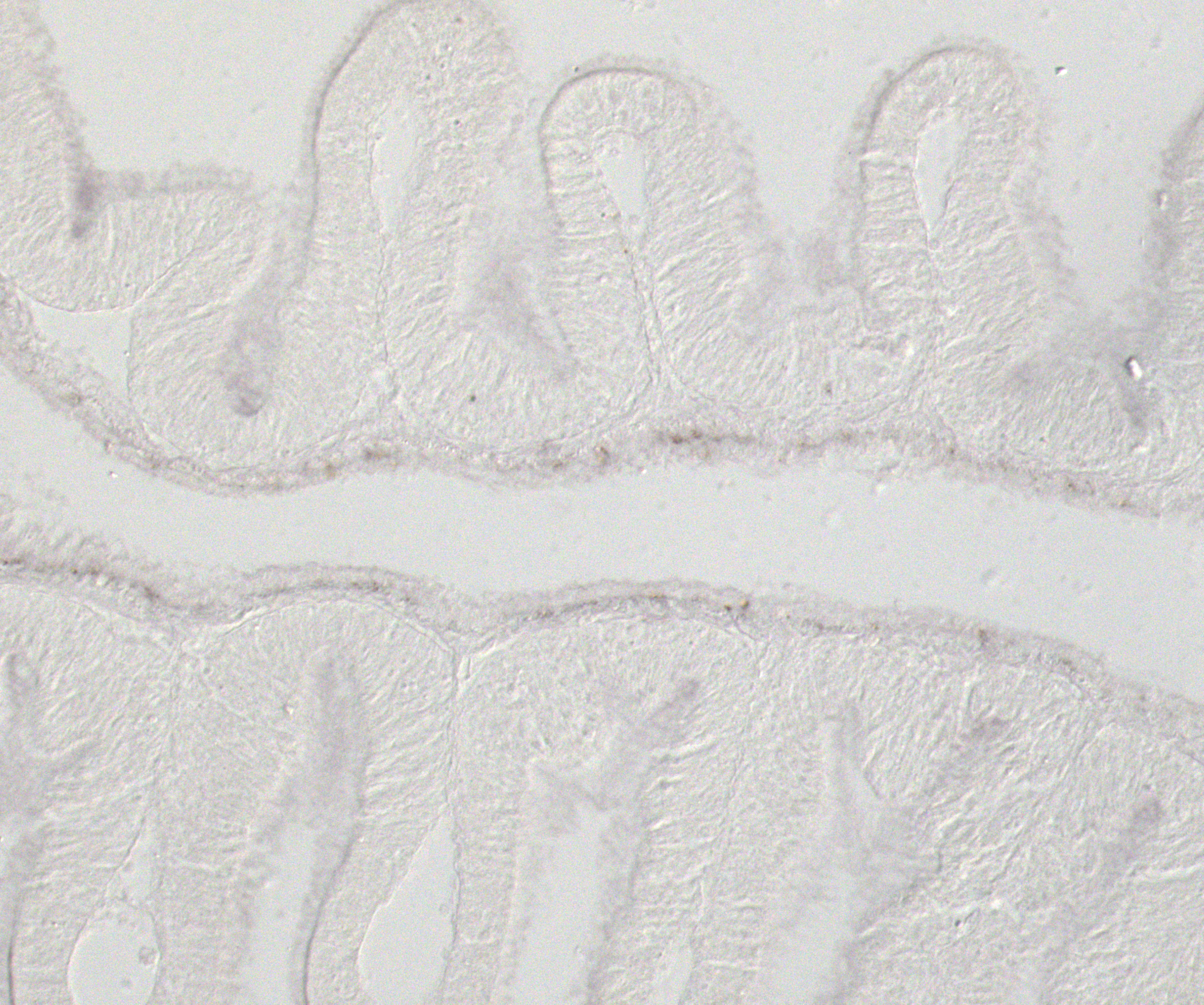

Supplement: Figure 8—source data 2. [file elife-101799-fig8-data2.zip › Figure 7-Source data 2/Figure 7H.tif]

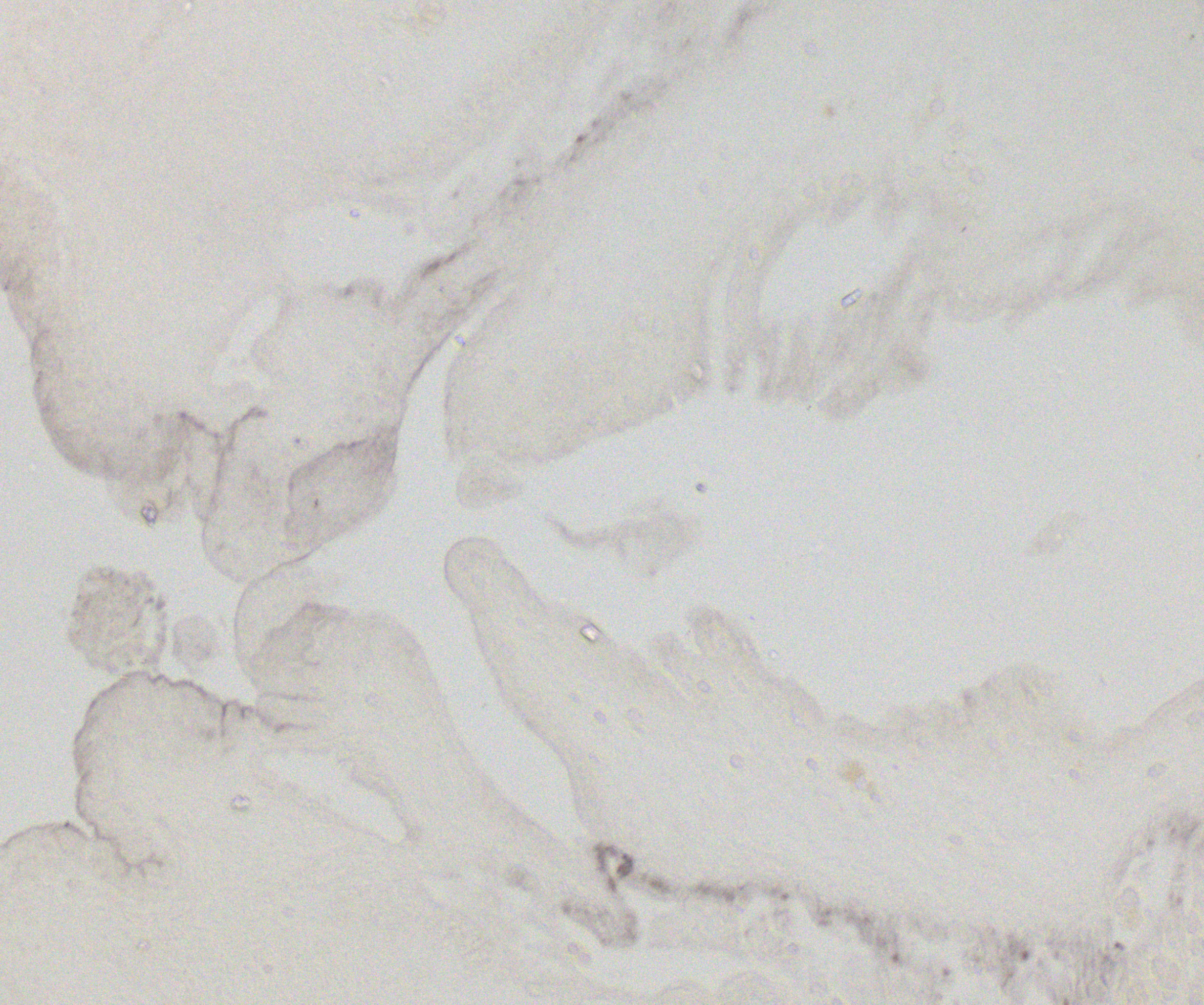

Supplement: Figure 8—source data 2. [file elife-101799-fig8-data2.zip › Figure 7-Source data 2/Figure 7I.tif]

**A**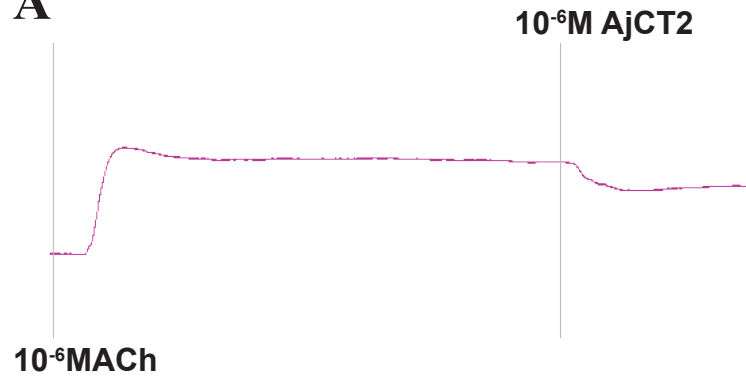**B**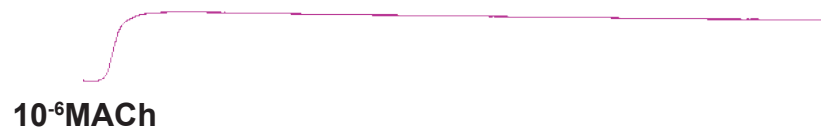**C**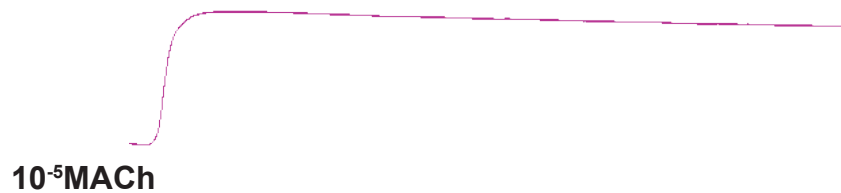

Supplement: Figure 8—figure supplement 1—source data 1. [file elife-101799-fig8-figsupp1-data1.pdf]
