# Supplementary material for: Functional characterization of neuropeptides that act as ligands for both calcitonin-type and pigment-dispersing factor-type receptors in a deuterostome
Source: eLife. 2025 Nov 21;13:RP101799. doi: 10.7554/eLife.101799 (PMC12638048; doi:10.7554/eLife.101799)
Supplement: Figure 5—figure supplement 1—source data 2. [file elife-101799-fig5-figsupp1-data2.pdf]

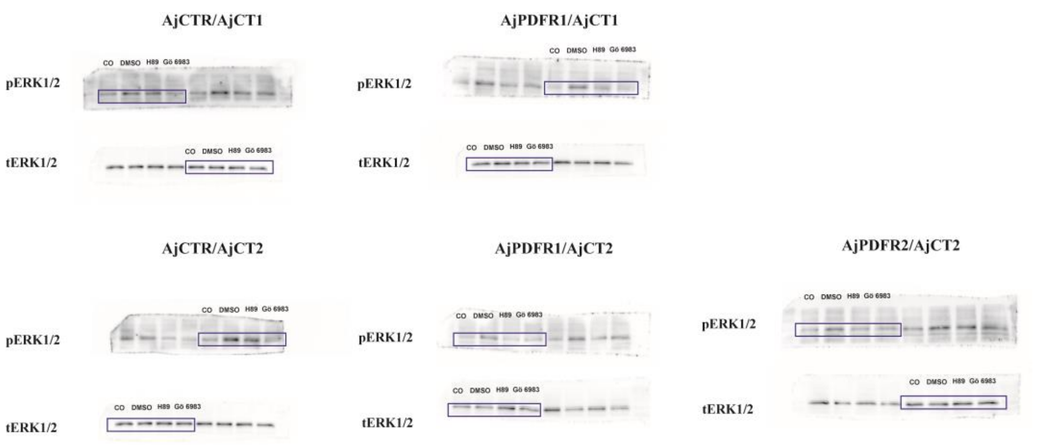

Figure 5-figure supplement 1-source data 2. Original membranes corresponding to Figure 5-figure supplement 1. The four blots outlined in blue are the target blots.
