# Supplementary material for: Functional characterization of neuropeptides that act as ligands for both calcitonin-type and pigment-dispersing factor-type receptors in a deuterostome
Source: eLife. 2025 Nov 21;13:RP101799. doi: 10.7554/eLife.101799 (PMC12638048; doi:10.7554/eLife.101799)
Supplement: Supplementary file 1. [file elife-101799-supp1.docx]

| **Reagent type (species) or resource** | **Designation** | **Source or reference** | **Identifiers** | **Additional information** |
| --- | --- | --- | --- | --- |
| sequence-based reagent | 5’-CGCGGATCCACAATGAGTGTCGGGAACGT-3’  5’-CCGGAATTCTCATTTCCCACCCCACTCAC-3’ | Custom synthesized by Tsingke |  | Oligonucleotide primers for pcDNA3.1(+) plasmid construction-- AjPDFR1 |
| sequence-based reagent | 5’-CGCGGATCCAAGATGGCGAACAATGAAAC-3’  5’-CCGGAATTCTCATTCATTAAATTTGCCGT-3’ | Custom synthesized by Tsingke |  | Oligonucleotide primers for pcDNA3.1(+) plasmid construction-- AjPDFR2 |
| sequence-based reagent | 5’-CCGGAATTCTGAAAATGCAGGAGAACGATTC-3’  5’-CGCGGATCCCGAACTACCGTTGTTTTATCTG-3’ | Custom synthesized by Tsingke |  | Oligonucleotide primers for pcDNA3.1(+) plasmid construction-- AjCTR–EGFP |
| sequence-based reagent | 5’-CCGGAATTCTGACAATGAGTGTCGGGAACGT-3’  5’-CGCGGATCCCGTTTCCCACCCCACTCACTGT-3’ | Custom synthesized by Tsingke |  | Oligonucleotide primers for pcDNA3.1(+) plasmid construction-- AjPDFR1–EGFP |
| sequence-based reagent | 5’-CCGGAATTCTGAAGATGGCGAACAATGAAAC-3’  5’-CGCGGATCCCGTTCATTAAATTTGCCGTTCA-3’ | Custom synthesized by Tsingke |  | Oligonucleotide primers for pcDNA3.1(+) plasmid construction-- AjPDFR2–EGFP |
| sequence-based reagent | 5’-AAGGTTATGCTCTTCCTCACGCT-3’  5’-GATGTCACGGACGATTTCACG-3’ | Custom synthesized by Tsingke |  | Oligonucleotide primers for qPCR assays *β-Actin* |
| sequence-based reagent | 5’-GAAAGCCTTACGACGGAACA-3’  5’-CACCACGTGGACTCAAAATG-3’ | Custom synthesized by Tsingke |  | Oligonucleotide primers for qPCR assays *β-Tubulin* |
| sequence-based reagent | 5’-GTTTGGCGGTACCCATTAC-3’  5’-GGCGTTCGGTCTGTTAATAG-3’ | Custom synthesized by Tsingke |  | Oligonucleotide primers for qPCR assays *AjCTP1/2* |
| sequence-based reagent | 5’-CGGTCCTTACTGTGCGGAGA-3’  5’-CGTCCTCCCAGCTGGACATC-3’ | Custom synthesized by Tsingke |  | Oligonucleotide primers for qPCR assays *AjMegf6* |
| sequence-based reagent | 5’-TGGAAGGCAGTAAGGCTA-3’  5’-TAGGCTGATGTGGTTGGA-3’ | Custom synthesized by Tsingke |  | Oligonucleotide primers for qPCR assays *AjIgf* |
| sequence-based reagent | 5’-ATACGCCCATACTCACATCC-3’  5’-TGGCAGGAGTCAACCTTCAT-3’ | Custom synthesized by Tsingke |  | Oligonucleotide primers for qPCR assays *AjGDF-8* |
| sequence-based reagent | 5’-GACGAATGGAGGAGAAGG-3’  5’-CGCCGCAATTAGAGTAGT-3’ | Custom synthesized by Tsingke |  | Oligonucleotide primers for qPCR assays *AjCTR* |
| sequence-based reagent | 5’-TTATCAACCGCCGTCTTC-3’  5’-CAGGAGGAGGAACACATTC-3’ | Custom synthesized by Tsingke |  | Oligonucleotide primers for qPCR assays *AjPDFR1* |
| sequence-based reagent | 5’-TGTTCTCCTGCCTCTTCT-3’  5’-GTCCTTCTCATCGGTGTG-3’ | Custom synthesized by Tsingke |  | Oligonucleotide primers for qPCR assays *AjPDFR2* |
| sequence-based reagent | 5’-GATTACGCCAAGCTTCGAGCTGTGTCGATAACATTACGCATGG-3’ | Custom synthesized by Tsingke |  | Oligonucleotide primer for 3’RACE *AjCTP1* and *AjCTP2* |
| sequence-based reagent | 5’-GATTACGCCAAGCTTGAGGGAAAGCCAAACCAGCGAGACG-3’ | Custom synthesized by Tsingke |  | Oligonucleotide primer for 3’RACE *AjPDFR1* |
| sequence-based reagent | 5’-GATTACGCCAAGCTTGTGCAACCGGACGAAGATCCACGTG-3’ | Custom synthesized by Tsingke |  | Oligonucleotide primer for 3’RACE *AjPDFR2* |
| sequence-based reagent | 5’-GATTACGCCAAGCTTCGTCTCGCTGGTTTGGCTTTCCCTC-3’ | Custom synthesized by Tsingke |  | Oligonucleotide primer for 5’RACE *AjPDFR1* |
| sequence-based reagent | 5’-GATTACGCCAAGCTTGCCTTCCACAAACATCCATGCGAACGC-3’ | Custom synthesized by Tsingke |  | Oligonucleotide primer for 5’RACE *AjPDFR2* |
| sequence-based reagent | 5’-GATTACGCCAAGCTTACGCCGGGCTCTCAAGAAGTCATTC-3’ | Custom synthesized by Tsingke |  | Oligonucleotide primer for 5’RACE *AjCTR* |
| sequence-based reagent | 5’-GGAAUUAUCAAGACAGUAU (dT)(dT)-3’  5’-AUACUGUCUUGAUAAUUCC (dT)(dT)-3’ | Custom synthesized by Tsingke |  | SiRNA1 for knocking down Aj*CTP* |
| sequence-based reagent | 5’-GAAAGUCGCUAAUGCAGUA (dT)(dT)-3’  5’-UACUGCAUUAGCGACUUUC (dT)(dT) -3’ | Custom synthesized by Tsingke |  | SiRNA2 for knocking down Aj*CTP* |
| sequence-based reagent | 5’-GUAGCGAUUGGUCGAAUAA (dT)(dT)-3’  5’-UUAUUCGACCAAUCGCUAC (dT)(dT)-3’ | Custom synthesized by Tsingke |  | SiRNA1 for knocking down *AjPDFR2* |
| sequence-based reagent | 5’-CAGUCAUUGUUAUCUACCA(dT)(dT)-3’  5’-UGGUAGAUAACAAUGACUG(dT)(dT)-3’ | Custom synthesized by Tsingke |  | SiRNA2 for knocking down *AjPDFR2* |
| sequence-based reagent | 5’-UUCUCCGAACGUGUCACGU(dT)(dT)-3’  5’-ACGUGACACGUUCGGAGAA(dT)(dT)-3’ | Custom synthesized by Tsingke |  | SiRNA for negative control |
